# 3D microfluidic gradient generator for combination antimicrobial susceptibility testing

**DOI:** 10.1038/s41378-020-00200-7

**Published:** 2020-11-02

**Authors:** Eric Sweet, Brenda Yang, Joshua Chen, Reed Vickerman, Yujui Lin, Alison Long, Eric Jacobs, Tinglin Wu, Camille Mercier, Ryan Jew, Yash Attal, Siyang Liu, Andrew Chang, Liwei Lin

**Affiliations:** 1 Department of Mechanical Engineering, University of California, Berkeley, CA 94720 USA; 2 Berkeley Sensor and Actuator Center, Berkeley, CA 94720 USA; 3 Department of Bioengineering, University of California, Berkeley, CA 94720 USA; 4 Department of Materials Science and Engineering, University of California, Berkeley, CA 94720 USA

**Keywords:** Chemistry, Engineering

## Abstract

Microfluidic concentration gradient generators (*µ*-CGGs) have been utilized to identify optimal drug compositions through antimicrobial susceptibility testing (AST) for the treatment of antimicrobial-resistant (AMR) infections. Conventional *µ*-CGGs fabricated via photolithography-based micromachining processes, however, are fundamentally limited to two-dimensional fluidic routing, such that only two distinct antimicrobial drugs can be tested at once. This work addresses this limitation by employing Multijet-3D-printed microchannel networks capable of fluidic routing in three dimensions to generate symmetric multidrug concentration gradients. The three-fluid gradient generation characteristics of the fabricated 3D *µ*-CGG prototype were quantified through both theoretical simulations and experimental validations. Furthermore, the antimicrobial effects of three highly clinically relevant antibiotic drugs, tetracycline, ciprofloxacin, and amikacin, were evaluated via experimental single-antibiotic minimum inhibitory concentration (MIC) and pairwise and three-way antibiotic combination drug screening (CDS) studies against model antibiotic-resistant *Escherichia coli* bacteria. As such, this 3D *µ*-CGG platform has great potential to enable expedited combination AST screening for various biomedical and diagnostic applications.

## Microfluidics: 3-D microfluidic gradient generator for antimicrobial susceptibility testing

Microfluidic concentration gradient generators (*μ-*CGGs) have been used to identify optimal drug compositions through antimicrobial susceptibility testing (AST) to treat antimicrobial-resistant infections, and a new method has been developed to overcome previous 2-D limitations. Conventional *μ-*CGGs fabricated through photolithography-based micromachining processes are limited to 2-D fluidic routing, which allows only two distinct antimicrobial drugs to be tested simultaneously. However, a team headed by Eric Sweet at the University of California, Berkeley, United States employed 3-D-printed microchannel networks that are capable of 3-D fluidic routing to generate symmetrical multi-drug concentration gradients. The authors were able to confirm their 3-D *μ-*CGG prototype by means of both theoretical simulations and experimental validations. The team believes that its 3-D *μ-*CGG platform offers considerable potential for conducting multi-drug AST evaluations for a variety of biomedical and diagnostic applications.

## Introduction

Treatment of antimicrobial-resistant (AMR) infections places a significant economic burden on the worldwide economy, upwards of $35 billion per year in the United States alone, and is projected to be the cause of over 10 million deaths per year by year 2050^1–3^. In the context of antibiotic resistance, for instance, more than 18 distinct bacteria, including pathogenic *Escherichia coli* (*E. coli*), *Listeria monocytogenes* (*L. monocytogenes*), and *Staphylococcus aureus* (*S. aureus*), have developed biological resistance to one or more of the world’s essential first-line-of-defense antibiotic agents^4,5^. The susceptibility of AMR organisms to antimicrobial compounds is assessed in clinical and biomedical research settings through antimicrobial susceptibility testing (AST) methods, predominantly minimum inhibitory testing (MIC) and combination drug screening (CDS), which are particularly useful in the fight against antibiotic-resistant infections, such as urinary tract infections (UTIs)^6^. Conventional MIC testing^7^ (Supplementary Materials Fig. S1a) involves overnight incubation of a patient-collected bacterial sample in the presence of growth media and dilute antibiotic solutions^8^ to determine the lowest dose of a single antibiotic required to inhibit the proliferation of bacteria and increase bacterial colony density, which is known as the MIC value^9,10^.

The MIC value therefore represents the lowest recommended antibiotic dose effective in treating a particular infection without encouraging further antibiotic resistance^11–13^. Furthermore, the only effective treatment against certain AMR infections is the use of combinations of multiple antimicrobial agents^14–17^. Conventional antibiotic CDS (Supplementary Materials Fig. S1b) is performed in a similar manner to MIC testing yet involves bacterial incubation in the presence of solutions containing specific ratios of different antibiotic compounds^8,18^ to ascertain the combined effects of the antibiotics, whereby certain *synergistic* antibiotic combinations are more effective at inhibiting bacterial proliferation than either of the two antibiotics on their own, while different combinations are either antagonistic, less effective, or additive, exhibiting neither combined effect^19,20^.

Conventional AST techniques, while well-established, generally require multiple independent manual labor-intensive fluidic handling procedures, involving a minimum of ~16–24 h for sample enrichment and dilution, followed by ~24–72 h for complete AST analysis^8,21^. As a result, the duration from sample collection to delivery of definitive AST results in clinical settings can take anywhere from 2 days to 1 week^10^. A standard clinical procedure while a clinician awaits AST evaluation, therefore, is to prescribe a large dose of a broad-spectrum antibiotic to stop the infection from worsening, which often contributes to the emergence and propagation of AMR in the first place^22–24^. Moreover, while MIC values^9,11^ and CDS results^16,20,25^ for specific antibiotics and bacterial strains can be found in the literature, antibiotic sensitivity can evolve over the lifetime of a bacterial colony^26^; therefore, frequent MIC and CDS testing is recommended in clinical settings^10,27,28^, posing a considerable limitation on AST throughput and overall cost, especially for screening more than two antibiotics at one time^29,30^.

Various microfluidic-based AST platforms^23,31^ have demonstrated miniaturized and multiplexed fluid handing^32,33^ to increase the throughput of AST analysis^29,34,35^ and decrease the mortality rate and healthcare costs^36,37^ associated with treating clinical AMR-related infections^38,39^, for novel drug development^40,41^, and for point-of-care clinical dosage recommendations^42–45^. Microfluidic concentration gradient generators (*µ*-CGGs), the most widely adopted class of microfluidic AST technologies^46^ for MIC and CDS studies, employ branching microchannel networks comprised of nodal units to produce diluted concentrations representing a gradient between input species. Discrete concentration gradients, for example, one antibiotic and one buffer solution^47,48^, are represented by the independent outputs from the *µ*-CGG network itself^49^, often referred to as discrete *µ*-drug cocktails^15^. Several discrete *µ*-CGG devices have been demonstrated for both single-antibiotic MIC testing and CDS studies^29,50^ of clinically relevant antibiotics against laboratory-standard bacteria^51^, bioengineered strains^52^, and cells isolated from biological fluid samples^40,50,53^. The *µ*-drug cocktail solutions are either used to perform on-chip bacteria culture^54^ or are collected as discrete approximately microliter-volume antibiotic solutions for use in off-chip bacteriological experiments^39^. As a result, a typical duration for rapid AST analysis is on the order of ~6–8 h^55^.

**Fig. 1:**
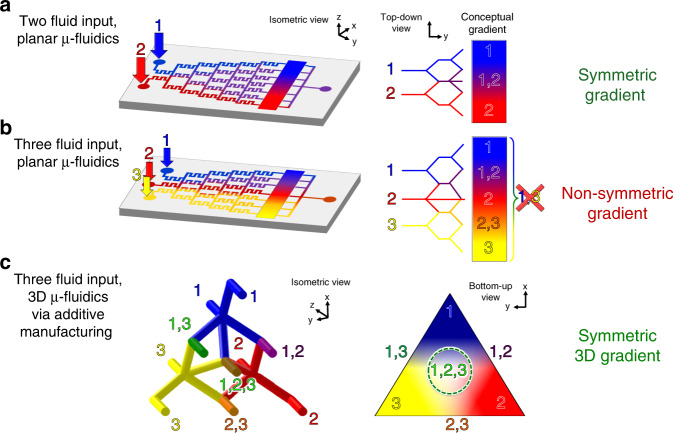
Conceptual microfluidic concentration gradient generators (*µ*-CGGs). **a** Conventional two-input planar devices (e.g., PDMS-based systems fabricated via soft lithography) generate symmetric gradients between both fluids, capturing all possible combinations of both species. **b** Limited to 2D fluidic processing due to the two-dimensionality of monolithic microchannel networks; such devices are unable to generate symmetric fluidic gradients between three or more inputs (i.e., no combinations of fluid inputs 1 and 3 are produced). **c** Only a truly 3D microchannel network capable of 3D fluidic routing, impossible to achieve using planar microfluidic fabrication methods, can generate symmetric 3D gradients of three or more input fluids (i.e., inputs 1 and 2, 2 and 3, 1 and 3, and 1, 2, and 3)

The application of *µ*-CGG enabled devices toward AST involving more than two antimicrobial compounds, however, is fundamentally limited. Traditional MEMS-based microfluidic fabrication approaches are monolithic in nature; as a result, conventional microchannels have inherently two-dimensional geometric complexity and are therefore capable of fluidic routing in essentially only two dimensions^56^. Symmetric fluidic gradients, those capturing all possible combinations of the inputs, are limited to only two distinct fluidic species at a given time (Fig. 1a). Previous *µ*-CGGs have demonstrated handling of more than two-input fluids; however, such gradients do not produce any combinations of nonadjacent fluidic species and are nonsymmetric (Fig. 1b). Alternative manufacturing approaches, including tedious and error-prone manual alignment and bonding of PDMS layers, have been demonstrated toward generating quasi-3D microfluidic structures; however, numerous limitations of such processes limit the 3D geometric complexity and practical functionality of such CGG designs^57,58^.

Given the ever-advancing capabilities, cost reduction and widespread commercial availability of high-resolution (≤100 µm) 3D-printing technology and additive manufacturing have garnered significant interest recently toward various microfluidic applications^59^; however, previously demonstrated 3D-printed microfluidic devices have fairly limited applicability towards AST applications, and, in particular, none have demonstrated the generation of discrete gradients of more than two antibiotics for AST^60–62^. Since conventional *µ*-CGG devices are therefore limited to producing *µ*-drug cocktails that capture the greatest range of possible combinations of only two antibiotics simultaneously, *µ*-CGG-enabled CDS of three or more antibiotics demonstrates significantly lower throughput and is fundamentally limited^29,63^.

This paper reports the design and development of a truly 3D *µ*-CGG prototype employing a unique 3D microchannel network that is only possible to fabricate using an additive manufacturing-based approach. The only way to accomplish a symmetric gradient of three or more fluids is to perform fluidic routing in truly three dimensions (Fig. 1c). A tetrahedrally arranged network of nodal microchannel units, geometrically symmetric in 3D space and capable of generating three inherently symmetric fluid gradients, was modeled and fabricated. The concentration gradient generation characteristics of the 3D *µ*-CGG were first theoretically simulated and used to optimize the design through the use of different integrated 3D microfluidic mixing (*µ*-mixer) structures to best elucidate the analytically predicted behavior; then, the performance of the fabricated prototype was experimentally validated through fluorescence imaging. Finally, antibiotic gradients collected from the device were used to demonstrate its proof-of-concept utility as an AST tool for MIC testing and pairwise and three-antibiotic CDS bacteriological experiments for three clinically relevant antibiotics against antibiotic-resistant *E. coli* bacteria.

## Results

### 3D *µ*-CGG design

**Fig. 2:**
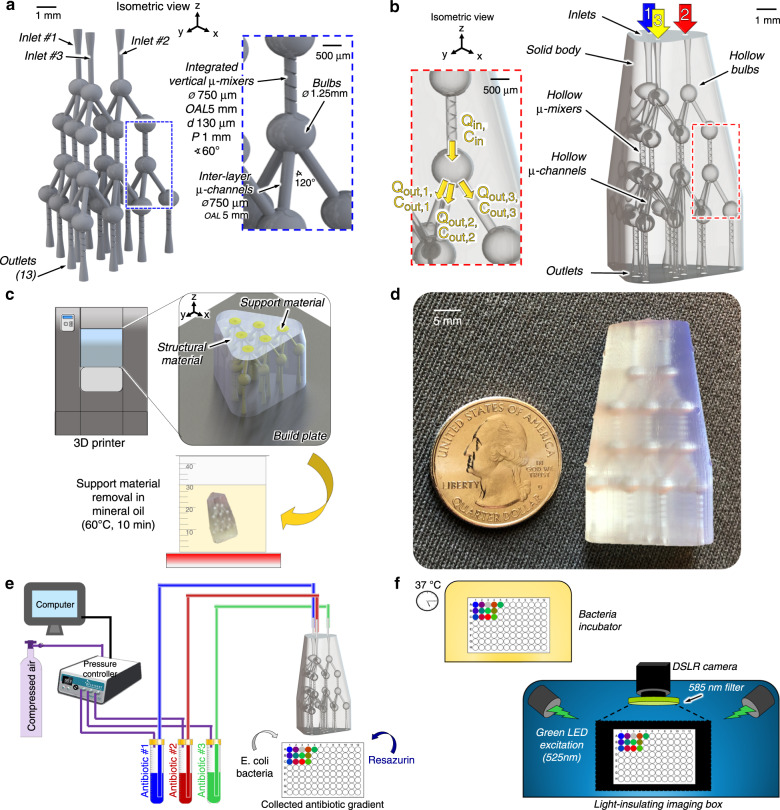
Developed 3D *µ*-CGG prototype and experimental setup. **a** 3D microchannel network design, dimensions, and an indication of labeling convention of device inlets 1, 2, and 3; positive solids model of all hollow structures comprising the truly symmetric 3D arrangement of nodal tetrahedral units (blue inset). **b** Reverse solids model, representing the manufacturable 3D microfluidic design comprising a single solid body with imbedded hollow microchannel structures; (red inset) flow rates (*Q*_in_, *Q*_out_) and input species concentrations (*C*_in_, *C*_out_) into and out of each nodal unit; these variables are used in all analytical device output calculations; fluid inputs indicated by colored arrows. **c** Concept of (top) 3D-printing fabrication and (bottom) postprocessing method to remove internal support material. **d** Fabrication results, actual 3D *µ*-CGG prototype after postprocessing; hollow interior structures are visible through the semitranslucent structural material, with US quarter for scale. Conceptual illustrations of the experimental setups to (**e**) collect the antibiotic gradient outputs from the fabricated 3D *µ*-CGG device and to (**f**) perform biological incubation and fluorescence imaging to quantify bacterial proliferation

The 3D *µ*-CGG microchannel network (Fig. 2a) features three fluidic input channels and 13 discrete output channels. The manufacturable design, consisting of microchannels as hollow structures in a solid body, is shown in Fig. 2b. Briefly, the network is comprised of a truly symmetric 3D arrangement of tetrahedrally arranged nodal combination–mixing–splitting units (Fig. 2b, c, insets). Fluids enter each nodal unit through a vertical channel, here shown with a channel-penetrating 3D-rifled microstructure (3D rifled *µ*-mixer) integrated into the sidewall, into a hollow spherical bulb (Supplementary Materials Sec. S1.3); then, the fluids flow symmetrically through independent outlets to the next nodal units. The network features three distinct layers of nodal units accomplishing truly symmetric fluidic routing in all three dimensions, generating equivalent proportions of the fluidic inputs to device inlets 1 & 2, 2 & 3, 1 & 3 and, most critically, 1, 2, & 3 at discrete outlets, which is impossible to achieve with planar fluidic routing. Comprehensive analyses of pressure-driven CGG networks using electric circuit analogies were published in seminal reviews in refs ^49,64^. Mathematical approaches traditionally used in the design of conventional 2D CGG’s^48,65^ were extended in this work to develop a nodal analytical methodology that was used to analytically calculate the expected output flow rates (*Q*_*i*_) and concentration of each input fluidic species (*C*_*i*_) at each nodal unit (Fig. 2b, inset) and device outlet (Supplementary Material Sec. S1.5). The fabricated and post processed (Fig. 2c) 3D *µ*-CGG prototype is shown in Fig. 2d, along with the experimental setups used to collect (Fig. 2e), incubate, and analyze (Fig. 2f) the output solutions. Further analysis of the resolution of the fabricated internal microstructures is presented in Supplementary Materials Sec. S4.

### Simulated concentration gradient generation performance

**Fig. 3:**
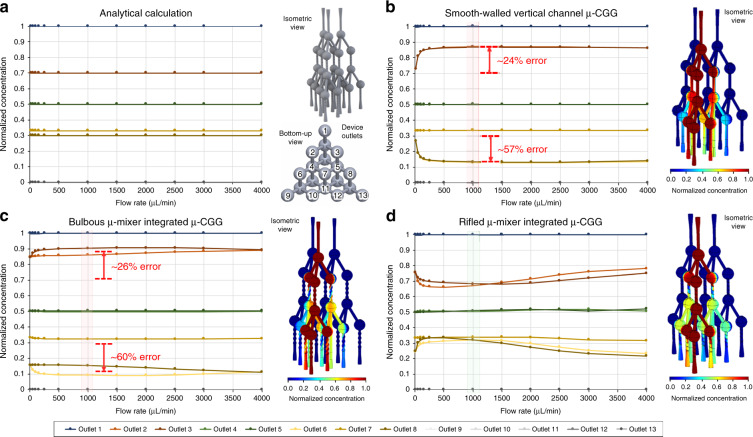
Theoretical performance of various 3D *µ*-CGG designs from COMSOL CFD simulations. **a** Analytical calculations for all 3D microchannel network designs are independent of the fluidic flow rate and outlet-numbering convention shown on the right and bottom. Theoretical results for (**b**) a design incorporating smooth-walled vertical channels, **c** a design incorporating vertical channels with repeating hollow spherical sidewall indentations, and **d** a design incorporating vertical channels with imbedded 3D rifling in the sidewalls. Plots (left) of normalized concentration of a single-input fluidic species from one of three device inlets at all 13 device outlets versus input fluid flow rate from 0 to 4000 µL/min, highest percent differences between analytical and simulated results are indicated in red, and the results at 1000 µL/min are also tabulated in Table 1; visualizations (right) of the concentration distribution on all surfaces of the positive solids model of the 3D microchannel network. The 3D rifled *µ*-mixer-integrated design in (**d**) exhibits the lowest error (within 10%) at all outlets and is the design chosen for prototyping, experimental characterization, and demonstration

One important assumption made during the analytical calculations of *Q*_*i*_ and *C*_*i*_ at each device outlet is that all fluids are completely mixed when they split off from a given nodal microchannel unit. The accuracy of the *Q*_*i*_ and *C*_*i*_ parameters of each fluidic output of the fabricated 3D *µ*-CGG prototype to the analytically calculated values is therefore highly dependent on the microfluidic mixing efficiency achieved by each nodal unit. To enhance the mixing quality inside each nodal unit, the effect of integrating intrachannel 3D *µ*-mixer structures into the sidewall of each vertical microchannel between the upper bulbs (where fluids combine) and the lower bulbs (where the fluids split off from) was investigated by studying three different 3D *µ*-CGG designs incorporating (i) smooth-walled vertical channels, serving as a reference, and imbedded (ii) 3D bulbous and (iii) 3D rifled microstructures, both based on a previously demonstrated 3D-printed *µ*-mixer^66^ to induce chaotic advective fluid motions for enhanced mixing efficiency^67^. The analytically calculated normalized concentrations (*N*_*c*_) of a single-input fluidic species (*C*_1_) at each outlet of a conceptual device (Fig. 3a) were independent of the input flow rate into the device. The theoretically predicted *N*_*c*_ values from the COMSOL simulation results for all 3D *µ*-CGG models over a range of symmetric input fluids flow rates from 0 to 4000 µL/min (Fig. 3b–d) become independent of input flow rate for all devices around ~1000 µL/min; therefore only the *N*_*c*_ values at *~*1000 µL/min were selected for comparison between devices. In addition, all analytical and theoretical results, and the percent errors between the two, are tabulated in Table 1.

**Table 1:** Theoretical results from COMSOL CDF simulations, the normalized concentration of a single-input fluidic species from one of three device inlets at all 13 device outlets (row 1) at an input flow rate of 1000 µL/min

Outlet #	1	2	3	4	5	6	7	8	9	10	11	12	13
Analytical	1.00	0.70	0.70	0.50	0.50	0.30	0.33	0.30	0.0	0.0	0.0	0.0	0.0
Rifled *µ*-mixer % error	1.00 0%	0.70 1.4%	0.70 2.9%	0.50 0%	0.50 2.0%	0.30 6.7%	0.33 6.7%	0.30 3.0%	0.0 0%	0.0 0%	0.0 0%	0.0 0%	0.0 0%
Bulbous *µ*-mixer % error	1.00 0%	0.86 22.8%	0.90 28.6%	0.50 0%	0.50 0%	0.09 70.9%	0.32 3.0%	0.15 50.0%	0.0 0%	0.0 0%	0.0 0%	0.0 0%	0.0 0%
Control channel % error	1.00 0%	0.87 24.3%	0.87 24.3%	0.50 0%	0.50 0%	0.13 56.7%	0.34 6.7%	0.13 56.7%	0.0 0%	0.0 0%	0.0 0%	0.0 0%	0.0 0%

Row 2: analytical calculations and theoretical results (top), with percent error from analytical values (bottom), for the (row 3) 3D rifled *µ*-mixer, (row 4) 3D bulbous *µ*-mixer and (row 5) smooth-walled vertical channel integrated 3D *µ*-CGG designs. Outlets with percent error higher and lower than 10% are indicated in red and green, respectively.

The reference *µ*-CGG design (Fig. 3b) exhibited the highest percent errors in theoretical *N*_*c*_ at device outlets 2 & 3 and outlets 6 & 8, with averages of ~24% and ~57% error, respectively, due to the inefficient microfluidic mixing inside the smooth-walled vertical microchannels. Furthermore, the 3D bulbous *µ*-mixer-integrated *µ*-CGG design (Fig. 3c) exhibited a comparable degree of inaccuracy at outlets 2 & 3 and outlets 6 & 8, with averages of ~26% and ~60%, respectively, which was likely still the result of incomplete fluidic mixing inside these particular 3D microstructure designs. The 3D rifled *µ*-mixer-integrated *µ*-CGG design (Fig. 3d), on the other hand, demonstrated the most accurate results, as the *N*_*c*_ values at all outlets are within 10% of the analytically calculated values. A conventionally accepted metric for the acceptable error of CGG output concentrations for AST applications is a maximum of 10%^39,49^. From this study, the 3D-rifled *µ*-mixer-integrated *µ*-CGG model was deemed capable of generating accurate (≤10% error) output concentrations representing practically useful proportions of each input fluidic species, i.e., ~1, *~*7/10, 5/10, ~3/10, and ~0, in addition to an output capturing a nearly equivalent proportion of all three inputs fluidic species, i.e., ~1/3, and was therefore chosen as the most appropriate design for prototype fabrication, experimental characterization, and bacteriological demonstrations.

### Experimental fluid flow characterization

The experimental characteristics of the fabricated 3D *µ*-CGG prototype were assessed by using the device to generate a gradient between one rhodamine fluorescent dye solution and two DI water fluidic inputs. Three experiments were performed with the rhodamine solution input into one of the device inlets, i.e., inlet 1, then the process was repeated with the rhodamine solution input into each of the other two inlets, i.e., inlets 2 and 3 (corresponding to the inlet labeling convention shown in Fig. 2a). With the device outputs collected on a 96-well plate, fluorescence imaging was used to measure the fluorescence emission of rhodamine in each solution in order to ascertain the distribution of the rhodamine solution from the device inlet of interest at every output of the device.

**Fig. 4:**
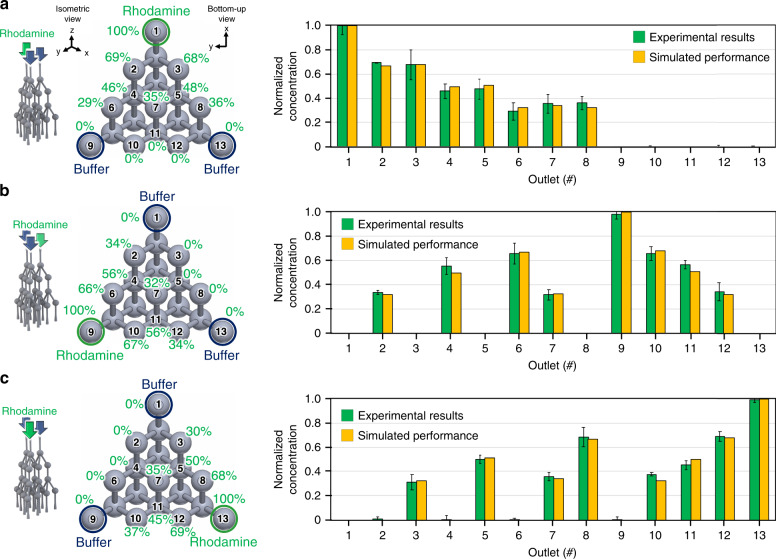
Experimental flow verification results validating the concentration of each fluidic input species at each device outlet. Normalized concentration of rhodamine fluorescent tracer dye input into (**a**) inlet 1, (**b**) inlet 2, and (**c**) inlet 3 (DI water input into the other two device inlets). Left: isometric view of the microchannel network and device input species and bottom-up view of the microchannel network outlets displaying the device outlet-numbering scheme; outlets containing 100% of each input species are indicated. Right: plot of the mean normalized rhodamine concentration (green) with theoretical COMSOL simulation results (yellow) for each outlet solution; error bars signify the standard deviation of triplicate experiments. Experimental results at all outlets are within 10% of the theoretically simulated values

With the rhodamine solution used as the input to inlet 1 (Fig. 4a), the mean experimental rhodamine *N*_*c*_ values of all device output solutions were within 10% of the theoretically predicted values from the COMSOL simulation results, exhibiting an average standard deviation of ~4.8%. Likewise, the distributions of rhodamine solution from inlets 2 (Fig. 4b) and 3 (Fig. 4c) among the device outlets produced *N*_*c*_ values within 10% of the theoretically predicted values as well, exhibiting average standard deviations of ~3.9% and ~3.1%, respectively. For further discussion, see Supplementary Material Sec. S7.3. Finally, the experimental *N*_*c*_ values from each experiment were used to quantify the concentration of each of the three fluidic input species contained in every device output solution, in terms of a percentage of the concentration of each input stock solution as presented in Supplementary Material Fig. S11. Regarding practical utility for three-fluid studies, the prototype demonstrated the ability to simultaneously generate three distinct gradients between only two of the three input fluidic species and produced at the outlets along each side of the bottom of the device (e.g., outlets 1, 3, 5, 8, and 13 capture a gradient between fluidic inputs to inlets 1 and 3 without contamination from that from inlet 2). Furthermore, 100% of each fluidic input was produced at outlets 1, 9, and 13; ~50% each of only two fluidic inputs were produced at outlets 4, 5, and 11; and an approximately equivalent proportion (average of ~34%) of each of the three fluidic inputs was produced from outlet 7.

### Bacteriological AST demonstrations

The ability of the developed 3D *µ*-CGG prototype to serve as a microfluidic platform for AST applications was demonstrated through proof-of-concept bacteriological experiments evaluating the antimicrobial efficacy of tetracycline, ciprofloxacin, and amikacin, three different clinically relevant antibiotic compounds commonly used to combat AMR-related infections^9^ (Supplementary Material Sec. S7.4.1), against an ampicillin-resistant strain of *E. coli* bacteria used as a demonstrative model. Briefly, all bacteriological experiments involved the generation of a gradient between antibiotic stock solutions and growth media using the fabricated *µ*-CGG prototype; the addition of metabolic indicator solution and *E. coli* inoculation to each discrete device output *µ*-drug cocktail solution collected on a 96-well plate, followed by incubation and detection. Resazurin salt (further discussed in Supplementary Material Sec. S6.1) was employed in these studies as a redox indicator of biological metabolism. Resazurin (dark blue in appearance) is readily metabolized to produce a bright pink molecule, resorufin, whereby the rate of resorufin production in solution is proportional to the rate of respiration of viable cells. Fluorescence microscopy is a well-suited method to detect resorufin production^68^, as resofurin is highly fluorescent (peak *l*_ex_ = 579 nm, *l*_em_ = 584 nm), whereas resazurin is weakly fluorescent. Resazurin-based cell viability protocols have proven simple, accurate, and reproducible methods to quantify and assess the metabolic activity of organisms, particularly bacteria^38,69,70^. In this work, normalized emission of resorufin was measured and used to produce a normalized growth (*N*_*g*_) value elucidating the degree of bacterial proliferation, and thereby the antibiotic-induced bacterial growth inhibition, in each solution^71,72^.

### Single-antibiotic MIC testing

**Fig. 5:**
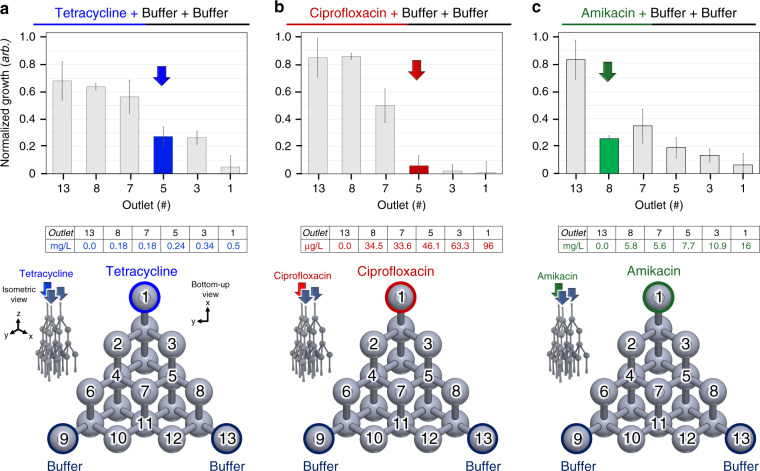
Experimental single-antibiotic minimum inhibitory concentration (MIC) testing bacteriological results, antibiotic solution input into device inlet 1, and growth media input into inlets 2 and 3 for all experiments. **a** Tetracycline, **b** ciprofloxacin, and **c** amikacin antibiotic MIC experiments, performed in triplicate. Top: mean normalized growth values and standard deviation error bars of fluid outputs capturing a gradient from 100% antibiotic to 100% buffer along one edge of the device, data representing the MIC value indicated by colored bars and calculated concentration. Middle: a table showing the concentrations of each of the antibiotics contained in the solution output from each device outlet. Bottom: illustration of the inputs into the microchannel network, device outlet-numbering convention and indication of the outlets containing 100% antibiotic and 100% buffer solutions

Experimental single-antibiotic MIC testing results are presented in Fig. 5. For each antibiotic, triplicate experiments were performed with the antibiotic solution into device inlet 1 and growth media solutions into inlets 2 and 3. Mean *N*_*g*_ values for solutions containing *µ*-drug cocktails from 100% antibiotic to 100% buffer are plotted from outlets along one edge of the device, i.e., outlets 1, 3, 5, 7, 8, and 13. The MIC value of the antibiotic was determined with the first apparent stepwise reduction in mean *N*_*g*_ value, i.e., enhancement in growth inhibition. The diagram in Supplementary Material Fig. S11a was then used to calculate the MIC value in mg/L. All experimentally determined MIC values, as well as MIC values from the literature, are presented in Supplementary Material Fig. S12.

Tetracycline has been one of the safest and most effective antibiotics used to treat serious conditions such as syphilis, cholera, malaria, and the plague and is also useful in multidrug treatments for AMR-related infections, such as bacterial peptic ulcers^20,73^. The tetracycline-buffer-buffer gradient (Fig. 5a) results indicated that the MIC value was represented by the *µ*-drug cocktail from outlet 5. It exhibited normalized growth of ~20% corresponding to a tetracycline concentration of ~0.26 mg/L, which is near the range for similar strains of *E. coli* in prior works^9,74^. The *µ*-drug cocktails containing higher concentrations of tetracycline than that from outlet 5 showed equivalent or lower amounts of growth, whereas those containing lower concentrations of tetracycline showed higher amounts of growth, between ~60% and 100%.

Ciprofloxacin is a commonly used antibiotic to combat UTIs, respiratory infections, and gastroenteritis and is often effective in combination with other antibiotics to treat AMR-related infections in CDS applications^16,25,30,75^. The experimental ciprofloxacin–buffer–buffer gradient (Fig. 5b) results indicated that the MIC value corresponded to the *µ*-drug cocktail from outlet 5. The *µ*-drug cocktail exhibited normalized growth of ~5%, corresponding to a ciprofloxacin concentration of ~50 µg/L, which is in agreement with the documented range for similar known multidrug AMR strains of *E. coli*^74,76^. Solutions containing higher concentrations of tetracycline showed roughly the same amount of growth, whereas those containing lower concentrations of ciprofloxacin show increased amounts of growth, ≤ ~50%.

Amikacin is a particularly effective antibiotic in combination treatments used to combat infections such as serious UTIs, tuberculosis and bacterial meningitis, and AST evaluation of amikacin is frequently performed in clinical and drug development settings^16,76–79^. The results from the amkacin–buffer–buffer gradient (Fig. 5c) study indicated that the MIC value was represented by the *µ*-drug cocktail from outlet 8. This cocktail exhibited normalized growth of ~30%, corresponding to a ciprofloxacin concentration of ~11 mg/L, which agrees well with the documented range of amikacin MIC values for similar strains of *E. coli*^20,74,79^. Solutions containing higher concentrations of amikacin showed equivalent or lesser growth, whereas the solution from outlet 13 containing no antibiotics showed the highest amount of growth.

### Pairwise antibiotic CDS studies

**Fig. 6:**
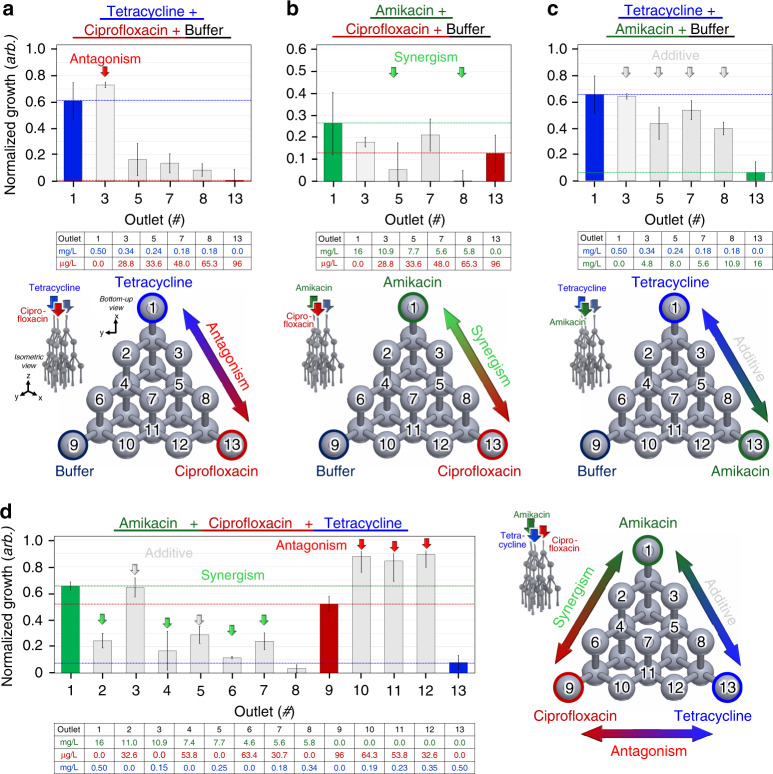
Experimental combination drug screening (CDS) bacteriological results revealing inhibitory interactions between all combinations of antibiotics, and experiments were performed in triplicate. *Pairwise* interaction studies with antibiotic input into inlets 1 and 3 and growth media input into inlet 2: **a** tetracycline + ciprofloxacin, antagonism (red arrow); **b** amikacin + ciprofloxacin, synergism (green arrow); and **c** tetracycline + amikacin, additive effect (gray arrow). Top: mean normalized growth values and standard deviation error bars of fluid outputs capturing a gradient between 100% of each antibiotic; data represent the known antibiotic interaction indicated by colored arrows. Middle: a table showing the concentrations of each of the antibiotics contained in the solution output from each device outlet. Bottom: illustrations of antibiotic inputs, device outlet-numbering convention, and an indication of outlets containing 100% of each antibiotic and buffer. **d** Three-antibiotic interaction study with antibiotic input into inlets 1, 2, and 3; (left) mean normalized growth values and standard deviation error bars of all 13 fluid outputs, data representing known antibiotic interactions indicated by colored arrows; (right) illustrations of the antibiotic inputs, device outlet-numbering convention and indication of outlets containing 100% of each antibiotic

The fabricated 3D *µ*-CGG prototype was also proposed for use as an AST tool to perform pairwise CDS studies. Triplicate experiments were performed with two different antibiotic solutions in device inlets 1 and 3 and growth media solution in inlet 2. Mean *N*_*g*_ values for solutions representing the gradient between 100% of both antibiotics collected from outlets 1, 3, 5, 7, 8, and 13 are plotted. The combined effects of each antibiotic pair follow similar trends from prior AST studies with related bacterial strains^16,25,45,80,81^. The specific ampicillin-resistant *E. coli* used in this work were experimentally assessed by comparing the *N*_*g*_ values of each solution, as shown in pairwise antibiotic CDS results in Fig. 6.

The gradient between tetracycline and ciprofloxacin (Fig. 6a) exhibited the expected antagonism^25^ in the solution from outlet 3 (~0.34 mg/L tetracycline, ~28.8 µg/L ciprofloxacin). This specific chemistry should therefore be avoided in treating an infection caused by this specific strain of bacteria. Furthermore, the gradient between amikacin and ciprofloxacin (Fig. 6b) demonstrated the expected synergism^16,45^ in the solutions from outlets 5 (~7.68 mg/L amikacin, ~48.8 µg/L ciprofloxacin), and 8 (~6.08 mg/L amikacin, ~65.3 µg/L ciprofloxacin); these specific chemistries are therefore be highly recommended in treating such an infection. Moreover, the gradient between tetracycline and amikacin (Fig. 6c) revealed the expected additive effect^80,81^ in all solutions from outlets 3, 5, 7, and 8; therefore, none of these solutions would serve as inherently beneficial treatments. All other solutions not explicitly mentioned demonstrated additive effects and are therefore not recommended as effective treatments.

### Three-antibiotic CDS assessment

Finally, the ultimate capability for AST evaluation of more than two antimicrobial agents was demonstrated using all three antibiotic solutions. Here, every single operation of the device generated three distinct pairwise gradients between amikacin and tetracycline (outlets 1, 3, 5, 8, and 13), amikacin and ciprofloxacin (outlets 1, 2, 4, 6, and 9), and ciprofloxacin and tetracycline (outlets 9, 10, 11, 12, and 13), in addition to the implementation of a three-antibiotic *µ*-drug cocktail, simultaneously.

The mean *N*_*g*_ data from the three-antibiotic CDS experiments (Fig. 6d) revealed the expected additive effect between amikacin and tetracycline (outlets 3 and 5), synergism between amikacin and ciprofloxacin (outlets 2, 4, and 6) and antagonism between ciprofloxacin and tetracycline (outlets 10, 11, and 12). Furthermore, an unexpected synergistic pairwise interaction was exhibited between amikacin and tetracycline from outlet 8. As a result, this specific chemistry would be recommended in treating an infection caused by the ampicillin-resistant *E. coli* strain, as demonstrated in this work. It should be noted that the metrics of three-antibiotic interactions are defined here as being relative to the interaction between any two of the antibiotics if they were to be combined in a two-antibiotic pair without the addition of the third antibiotic. As a result, the *µ*-drug cocktail containing a nearly equivalent proportion of all three input antibiotic compound concentrations (outlet 7) demonstrated synergism compared to the 100% solutions of amikacin and ciprofloxacin and an additive effect compared to the 100% solutions of amikacin and tetracycline, as well as ciprofloxacin and tetracycline. In this demonstration, the *µ*-drug cocktails from outlets 2, 4, 5, 6, 8, and 13 exhibited more effective inhibition of bacterial proliferation than those of the three-antibiotic *µ*-drug cocktail and would, therefore, represent more effective doses for treatment. Regardless, the three-antibiotic *µ*-drug cocktail is the direct result of the unique 3D fluidic routing capability demonstrated by the 3D *µ*-CGG microchannel network, which is otherwise impossible using planar fluidic routing processes.

## Discussion

The throughput of multidrug AST applications using conventional *µ*-CGG devices is fundamentally limited by the inability of such systems to generate symmetric concentration gradients of more than two antimicrobial solutions at a time. In this work, we developed a 3D *µ*-CGG prototype employing a truly 3D microchannel network through the use of an additive manufacturing approach to accomplish fluidic routing in three dimensions to generate symmetric three-fluid concentration gradients.

Analytical modeling and theoretical simulations were used to design and optimize the microchannel network via the inclusion of embedded 3D *µ*-mixing structures to produce 13 distinct output *µ*-drug cocktail solutions for bacteriological studies. Experimental characterizations validated the generated concentrations to within 10% of the predicted values, justifying the use of the proposed 3D *µ*-CGG system for multidrug AST evaluations.

As a proof-of-concept, the fabricated prototype was used to evaluate the efficacy of three clinically relevant antibiotic compounds against model antibiotic-resistant *E. coli* bacteria. The MIC values of the individual antibiotics were characterized and were in agreement with the documented ranges for each compound. Furthermore, the known synergistic, additive, and antagonistic effects of each combination of antibiotics were experimentally observed through individual pairwise CDS studies.

As such, the unique 3D fluidic routing capabilities enabled a three-antibiotic CDS study to simultaneously generate three distinct pairwise antibiotic concentration gradients, including one *µ*-drug cocktail containing all three antibiotic species, in 5 h from a single operation for enhanced throughput over conventional multiantimicrobial CDS approaches.

Moving forward, additive manufacturing permits straightforward and on-demand modification of 3D *µ*-CGG designs to produce tailored concentration gradient characteristics. Such devices can be rapidly prototyped and fabricated in clinical point-of-care settings using commercially available 3D printers to reduce the time-to-deployment and manufacturing costs. Ongoing technological advances in additive manufacturing resolution, material variety, and scalability will enable engineering of evermore advanced 3D *µ*-CGG designs that can be incorporated into more complex micrototal analytical systems to significantly increase the throughput of AST to combat emerging antibiotic-resistant bacterial infections in clinical and drug development settings.

## Materials and methods

### Design of the 3D microchannel network

All microfluidic designs were modeled using Solidworks computer-aided design software (Dassault Systemes, Velizy-Villacoublay, France). All inlet and outlet geometries enabled device-to-world interfacing via standard 20-gauge stainless-steel catheter couples (SC20/15, Instech, PA, USA). All microchannels, bulbs, and solid bodies were saved as individual part files then arranged in a single assembly file to produce the positive-feature network (Fig. 2a) for use in all theoretical simulations. Subtraction of the model with a solid body was used to produce the final manufacturable design (Fig. 2b), saved as a single part file, exported as an.STL file and imported to 3D-printing software for manufacturing.

### Prototype fabrication and postprocessing

Additive manufacturing of the prototype was accomplished using a Projet 3000UHD Multijet modeling 3D printer (3D Systems, SC, USA) (Fig. 2c). The materials employed in this work were Visijet M3 crystal polymer^82^ (3D Systems) and Visijet S100 hydroxylated wax^83^ (3D Systems). Both materials (Supplementary Material Sec. S2.1) were deposited simultaneously in an inkjet-like process in ~35-µm thick layers with a lateral feature resolution as low as 50 µm^84^. The support material, which was necessary to reinforce and successfully resolve overhanging geometries such as the 3D *µ*-mixer rifling and hollow spherical cavities, was removed from the device after 3D printing using a previously demonstrated postprocessing protocol^66,85^. Briefly, the prototype was placed inside a preheated oven at 75 °C for 15 min with the outlets facing downwards on top of paper towels to facilitate drainage of support material from the microchannels through capillary action. The device was then submerged in a beaker containing food-grade Bayes mineral oil preheated to ~60 °C for ~10 min. The heated mineral oil was flushed through each device outlet three times using a 10-mL syringe (Cole-Palmer, IL, USA) attached to a 20-Gauge Luer stub (model LS20, Instech) until all of the interior support material was removed. Finally, the process was repeated using an aqueous soap solution and potable water to remove any residual mineral oil (Fig. 2d). For further details, see Supplementary Material Sec. S2.3.

### Theoretical simulations

Computation fluid dynamics (CFD) simulations were performed using COMSOL Multiphysics (Version 4.5a, COMSOL, Inc., CA, USA) finite element analysis software to determine the theoretical performance of all 3D models. The theoretically predicted normalized concentration (*N*) of the species from one inlet (*C*_1_) at each outlet for a range of input fluid flow rates was calculated as the average *N* value over the entire microchannel outlet face (Fig. 3). Due to the 3D symmetry of all simulated microchannel networks, and the *N* values for *C*_1_ assigned to device inlet 1 were identical to the results if *C*_1_ was assigned to inlets 2 and 3. Therefore, in this work, only *C*_1_ assigned as the inlet 1 input was simulated. For further details, see Supplementary Material Sec. S5.

### Preparation of cell solutions

Ampicillin-resistant BL21(DE3) Gram-negative *Escherichia coli* (*E. coli*) bacteria were procured from Agilent Technologies, CA, USA^86^, in 2-mL cryovials as glycerol stock and stored at −80 °C. A scraping from the glycerol stock was added to 10 mL of Lysogeny Broth (LB media), a bacteria-specific nutrient-rich solution, in a 25-mL T25 flask with a breathable filter cap (#169900, ThermoFisher Scientific, MA, USA) and enriched in a bacteria incubator at 37 °C and 4% CO_2_ overnight.

The following day, ~10 µL of the solution was transferred to agar media to create a solid bacteria colony plate^10^, which was stored at −4 °C for use for up to 1 month. Before each day of bacteriological experiments, a fresh bacterial inoculation was created by adding a single bacterial colony harvested from the agar plate to 10 mL of LB media and incubating overnight at 37 °C. The cell density of the solution was measured the next day using a UV-VIS spectrophotometer (Vernier, OR, USA) following the OD600 method^8,87^; then, a serial dilution was performed to create an inoculation with an initial cell density of ~5 × 10^5^ cfu/mL following cell viability^88–90^ and AST^38,50,52,69,91^ conventions. LB media was prepared by dissolving LB powder (LB Miller Broth, Sigma-Aldrich, MO, USA) in deionized (DI) water to make a 25 g/L solution in a 1000-mL autoclave bottle (ThermoFisher Scientific), followed by autoclave sterilization at 120 °C for 25 min. For further details, see Supplementary Material Sec. S6.2.

### Preparation of reagents and antibiotic solutions

Whenever solids were added to liquids or multiple solutions were combined to create new solutions, sterile 50-mL conical polypropylene centrifuge tubes (#339652, ThermoFisher Scientific) were used, aggravated using a vortex mixer (Vortex-Genixe 2, Scientific Industries, NY, USA) for ~30 s to ensure a homogeneous solution, and then wrapped in aluminum foil and stored at −4 °C for up to 1 week of use. Rhodamine fluorescent dye solution was created by adding 50 drops of 0.04% fluorescent red dye Rhodamine B solution (OnlineScienceMall) to 50 mL of DI water. Resazurin metabolic indicator solution was prepared to a concentration of ~4.4 mM by adding ~5 mg of resazurin salt powder (Sigma-Aldrich) to 50 mL of sterile LB media. Antibiotic solutions were prepared by combining the required mass of antibiotic powder (each acquired from Sigma-Aldrich) to 500 mL of sterile LB media to create the desired antibiotic stock solution concentration, roughly twice the approximate average of published MIC values for a specific antibiotic against *E. coli*. For all concentrations of antibiotic stock solutions used throughout this work, see Supplementary Material Fig. S12.

### Device operation and collection of output gradients

Discrete fluidic outputs from the fabricated 3D *µ*-CGG prototype were collected using the experimental setup conceptually illustrated in Fig. 2e. Briefly, for each experiment, fluids from three independent fluidic reservoirs attached to a MAESFLO microfluidic control platform (Fluigent, Paris, France) were pressure-driven through the three device inlets at a steady volumetric flow rate of 1000 µL/min for one minute. The device output solutions, each contained in discrete segments of Tygon tubing (#06420-03, Cole-Palmer, IL, USA) cut to length to isolate the desired volume, were routed to discrete wells on a standard microwell plate (ThermoFisher Scientific). See Supplementary Material Sec. S7.1 for further details.

### Experimental fluid flow characterization

The 3D *µ*-CGG device was used to generate gradients between one rhodamine fluorescent dye solution and two DI water inputs, and 90 µL of output solution was collected on a 96-well plate. Briefly, using the fluorescence imaging setup (Fig. 2f), the normalized concentration of rhodamine in each solution, averaged over three experiments, was determined by imaging each solution under UV light excitation inside a custom-built light isolation box with a red bandpass optical filter (610-700 nm, #W6308, Omega Optical, VT, USA) and DSLR camera (Canon EOS 1000D, Canon, Tokyo, Japan), and the excitation and emission peaks of rhodamine were set as 540 nm and 625 nm, respectively^92^. Rhodamine fluorescence emission in each solution was quantified using the image analysis protocol detailed in Supplementary Material Sec. S7.3.

### Bacteriological AST experiments

For each experiment, the 3D *µ*-CGG device was used to generate gradients between antibiotic and buffer solutions. The output (30 µL) *µ*-drug cocktail solutions were collected on a 96-well plate, then 30 µL of each resazurin metabolic indicator solution and *E. coli* inoculation were pipetted into each well. A 0% antibiotic (fully uninhibited proliferation) control consisting of 30 µL each of bacterial inoculation, resazurin solution, and LB media was pipetted into one well, and the no-proliferation control, consisting of 30 µL of resazurin solution and 60 µL of LB media, was pipetted into another well. For further details, see Supplementary Material S7.4. Each plate was first incubated at 37 °C (Fig. 2f) until a visible gradient between blue and pink from the 0% antibiotic control across the device outputs was observed for an average of ~5 h. The normalized bacterial proliferation in each solution, averaged over three experiments, was determined by imaging each solution under excitation from green LED light^69^ using a 585-nm optical filter (#W6308, Omega Optical)^93^. The fluorescence emission of resorufin in each solution was quantified following the image analysis protocol detailed in the Supplementary Material Sec. S7.4.

## Supplementary information

Supplementary Materials: 3D Microfluidic Concentration Gradient Generator for Combination Antimicrobial Susceptibility Testing
