# Supplementary material for: 3D microfluidic gradient generator for combination antimicrobial susceptibility testing
Source: Microsyst Nanoeng. 2020 Nov 2;6:92. doi: 10.1038/s41378-020-00200-7 (PMC8433449; doi:10.1038/s41378-020-00200-7)
Supplement: Supplementary file 1 — Supplementary Materials: 3D Microfluidic Concentration Gradient Generator for Combination Antimicrobial Susceptibility Testing [file 41378_2020_200_MOESM1_ESM.pdf]

Supplementary Materials:

3D Microfluidic Concentration Gradient Generator for Combination Antimicrobial Susceptibility Testing

Authors:

Eric Sweet[1,2*], Brenda Yang[2,3], Joshua Chen[2,3], Reed Vickerman[1,2,4], Yujui Lin[2], Alison Long[2,3], Eric Jacobs[2,3], Tinglin Wu[2,3], Camille Mercier[2,3], Ryan Jew[1,2,3], Yash Attal[2,3], Siyang Liu[1,2], Andrew Chang[2], and Liwei Lin[1,2]

Affiliations:

[1] Department of Mechanical Engineering, University of California, Berkeley, 94720, USA

[2] Berkeley Sensor and Actuator Center, Berkeley, 94720, USA

[3] Department of Bioengineering, University of California, Berkeley, 94720, USA

[4] Department of Materials Science and Engineering, University of California, Berkeley, 94720, USA

*** To whom correspondence should be addressed:**

Eric Sweet

E-mail: ericsweet@berkeley.edu

Liwei Lin

E-mail: lwlin@berkeley.edu

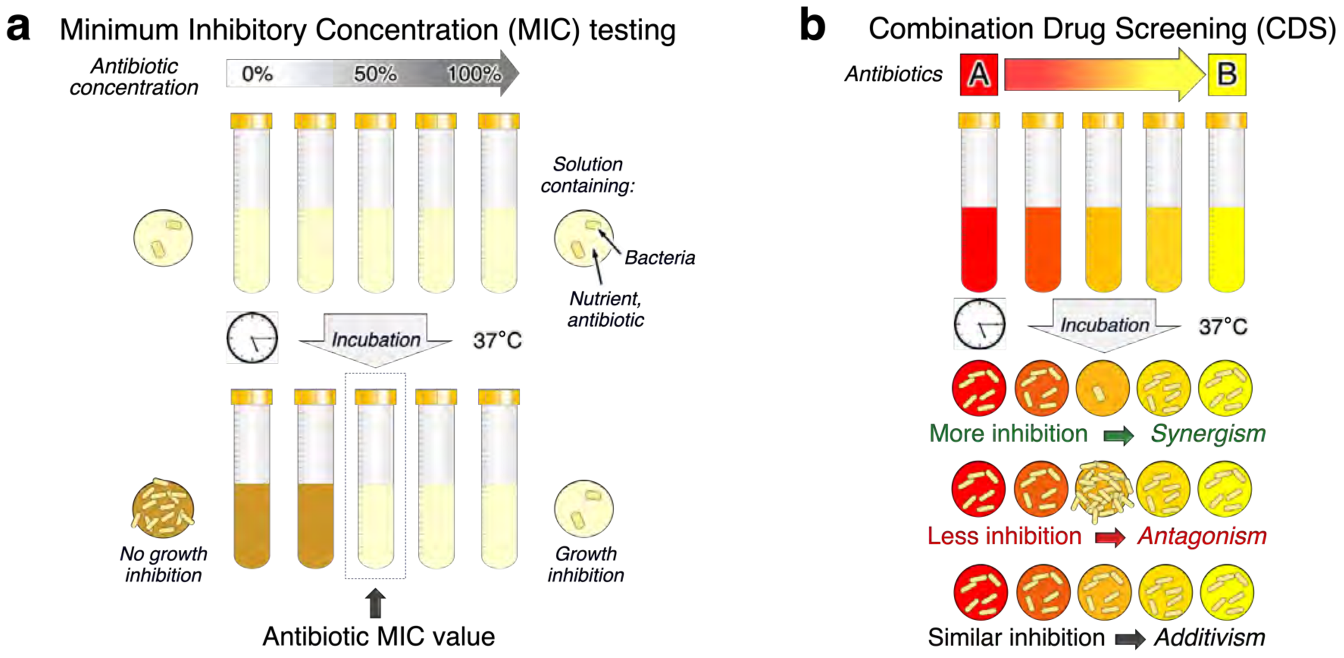

Figure 1. Conceptual gold-standard antibiotic susceptibility testing (AST) protocols via conventional broth dilution methods[1] involving incubation of bacteria, nutrient and antibiotic solutions. **(a)** Minimum Inhibitory Concentration (MIC) testing to determine the lowest concentration of a single antibiotic required to inhibit the proliferation of bacteria. **(b)** Combination Drug Screening (CDS) to determine the combined effects of multiple antibiotics on the inhibition of bacteria proliferation.

1 3D μ-CGG Design

1.1 General 3D μ-CGG design approach via additive manufacturing

In light of the fundamental limitations of conventional 2D μ-CGG devices inherent to their planar manufacturing methods, the objective of this project is to design, fabricate and demonstrate *three-dimensional* μ-CGG structures capable of generating truly *symmetric* multi-fluid gradients. The only way of accomplishing such a symmetric gradient of three or more fluids is to utilize the *third spatial dimension* and perform fluidic routing and mixing in a truly-*three-dimensional* fashion, a task which is fundamentally hindered by the limitations of conventional planar fabrication methods. As a means to this end, this work demonstrates the use of a Multijet 3D printing to fabricate entirely-3D printed 3D μ-CGG prototypes which utilize unique *three-dimensionally*-arranged microchannel networks only possible to fabricate *via* an additive manufacturing-based fabrication approach. As Figure **??**a illustrates, by utilizing the additive manufacturing nature of 3D printing, the *third-spatial dimension* is now available in which to fabricate microchannel structures that perform the 3D fluidic routing necessary to create combinations of fluidic species input to Inlets #1 & #2, Inlets #2 & #3, Inlets #1 & #3, and most critically, Inlets #1, #2 & #3 of the device. As a result, the conceptual gradient of all three input fluidic species is inherently *symmetric*, as all possible combinations of all three input fluidic species are generated, simultaneously.

1.2 Integrated μ-mixing structures

The concept of intra-channel microstructures (μ-mixers) intended to enhance microfluidic mixing efficiency has been previously studied[2,3]. Our group has previously developed new classes of entirely-3D printed, 3D intra-channel chaotic μ-mixers for low Reynolds Number microfluidic applications, designs which can be incorporated into arbitrary locations inside microchannels where enhance microfluidic mixing is desired[2]. The lead authors of this paper are currently preparing a manuscript for consideration to peer reviewed journals detailing further development and analysis of such designs. This work features a simplified version of our previously-developed 3D rifled μ-mixer structure. The design features a microchannel with a nominal inner diameter of 750 μm and overall length (OAL) of 5mm with a rifling structure which penetrates into the fluid flow with 3D geometric complexity. The rifled structure is an equilateral triangle in cross section with side lengths of 130 μm, a pitch of 1mm and an internal angle of 60 degrees. Four complete periods of rifling provide for enhanced chaotic mixing of co-laminar microfluidic flows at the outlet of the vertical microchannel, as compared to co-laminar flow under the same conditions flowing through a vertical channel with smooth side walls. See our publication in Ref.[2] for theoretical and experimental validation of such a 3D μ-mixer design.

1.3 Hollow spherical microchannel bulbs

Each nodal microchannel unit is comprised of an upper hollow bulb where multiple fluids enter and are combined, a vertical microchannel with integrated μ-mixing structures, and a lower hollow bulb where the fluid flow is then symmetrically-split *via* a tetrahedron shape-based arrangement of microchannels at equivalent angles (120 °). The bulbs featured in this design are 1.25mm in diameter. One reliable method of enhancing co-laminar inter-fluid species mixing is to induce non-normal, or transverse, fluid velocities in the co-laminar flow, using either 2D or 3D intra-channel structures, in order to induce chaotic fluid advection[4]. With this principle in mind, the intent behind including the hollow bulb in each microchannel nodal unit was to induce transverse fluid velocities in the incoming fluids which combine in the upper bulb as the fluids flow outwards along the side-wall of the hollow bulb, such that when they recombine before entering the 3D rifled μ-mixer integrated microchannel, the transverse fluid velocities in the co-laminar flow would increase the degree of chaotic advection in the fluid even before the 3D rifling acts on the fluid flow, inducing further fluidic mixing. Without the inclusion of the upper hollow bulbs, the nodal unit fluidic input channels could combine directly to form co-laminar flow at the inlet to each vertical microchannel with integrated μ-mixing structures.

Additionally, the intent behind utilizing a lower hollow bulb used in each nodal microchannel unit was to induce a pressure drop the output fluid from the μ-mixer integrated vertical microchannel, thereby expanding the surface area and reducing the velocity of the fluid flow, permitting more uniform fluidic routing through the multiple fluidic outlets which split off from the nodal unit and serve as the inlets to the subsequent nodal units in the microchannel network. Without inclusion of the lower hollow bulbs, it was theorized that more uneven fluid flow division would occur, thereby causing unintentional error propagation in concentration gradient generation performance.

During development and characterization of the fabricated prototypes, however, additional unintentional effects on fluid flow throughout the microchannel network induced by the hollow bulbs were considered. For example, the large hollow regions in the microchannel network provide hollow cavities for the likely non-zero potential for support material to remain trapped inside during post-processing, due to the pressure drop experienced by the forward pressure-driven mineral oil as it flows throughout the fluidic network in order to flush out the melted support material. As a result, the remaining support material residue, non-uniformly coating the interior of each hollow bulb, would change the inherent dimensions of the microchannel, increasing the asymmetry of the 3D microchannel arrangement and fluid flow patterns inside each hollow bulb, thereby leading

to unintentional fluid flow patterns and overall concentration distribution at the device outlet, as predicted by by both analytical calculations and theoretical simulations. In related work towards the development of 3D printed fluidic circuitry[5], this potential issue was addressed by including temporary access inlets to the most potentially problematic hollow regions, which provide direct access to apply higher oil pressures to the given hollow bulb, thereby increasing the likelihood of efficient support material removal. In this instance, following post-processing, these inlets are permanently sealed using epoxy to prevent fluid leaking from the device. In order to enable more effective and efficient support material removal and increase the reliability of fluidic mixing and predictable gradient generation, improved 3D μ-CGG designs can utilize a similar approach by including such sacrificial access inlets to complex microchannel networks.

Furthermore, the hollow volume of the hollow bulb structure itself, even if perfectly absent of support material residue in the theoretical case, could also provide for localized fluid flow disturbances or air bubbles to be trapped inside the fluidic network during operation, which would likely also contribute to unintentional alteration of the fluid dynamics and propagating errors in fluid flow further down in the fluidic network, resulting in an unintentional concentration distribution at the device outlets. Further study of various hollow bulb volumes and geometries, or potentially the removal of the hollow structures themselves, could help to shed light on the various effects on fluid flow that different geometries have during the design and development phase of future 3D μ-CGG networks to improve the accuracy of concentration gradient generation.

1.4 Layer-based nodal unit arrangement

The nodal units are arranged into what can be defined as two tiers, or layers. After the first layer, the nodal units which produce (∼50%) of each pair of inlet fluidic species, in addition to routing the combined fluid flow to the next layer, also consist of a long vertical microchannel, termed the *dropdown outlet*, which generates a collectable-fluidic outlet. A more detailed description of the layered nodal unit arrangement, as well as a derived design equations which are used in the analytical calculations of output fluid flow rates and concentrations at each outlet are presented in Figure S2.

1.5 Analytical nodal analysis and calculation of output flow rates and concentrations

Due to the symmetry in the 3D microchannel network geometry (*i.e.* the fluid paths between device Inlets #1 & #2 to each device outlet are identical to those between Inlets #1 & #3 and Inlets #2 & #3), nodal analysis calculations were performed along the fluidic paths between Inlets #1 & #2, and at any location where contribution from the species from Inlet #3 was present, the concentration of that species was determined from the reflection of the geometry over the plane of geometric symmetry and the value that was used was the value of the species from Inlet #2.

Briefly, each location in the 3D fluidic network design where fluids combine and split from a hollow bulb is treated as an individual *nodal unit*. At this point, significant assumptions are made in the nodal analysis. The assumption is made that all fluids that enter the upper hollow bulb from the input microchannels and flow through the vertical microchannel are homogeneously mixed by the time that they enter the lower hollow bulb and split off and exit at equivalent flow rates through all of the outlet microchannels. By making this assumption, the group consisting of the upper and lower hollow bulbs and vertical microchannel of each nodal unit can be treated as a control volume and nodal analysis can then be performed using the following calculations and analytical steps in order to determine the fluid flow rate and species concentrations of the input fluids to, and output fluids from, each control volume. Nodal analysis proceeds from the top of the microchannel network (*i.e.* at the fluidic inlets) to each subsequent nodal unit, and ending at the bottom of the network (*i.e.* at the fluidic outlets) where the final values are calculated and reported (*i.e.* the total fluidic flow rate, Q, and the total concentration of each input species, C_1, C_2 & C_3, contained in the fluidic volume),

Nodal analysis begins at the two outer-most hollow bulbs on the first layer for both designs. The flow rate and concentration of the input fluidic species into each initial nodal unit are known (Q_1 and C_1 into Inlet #1; Q_2 and C_2 into Inlet #2). Each of these control volumes therefore sees a single input and multiple outputs. Each of the fluid output therefore contains 100% concentration of the input fluidic species and, for example, have flow rates of exactly one half of the input flow rate. For all subsequent control volumes with multiple fluidic inputs, the following nodal analysis procedure is performed.

First, the hydraulic resistances of each of the microchannels exiting the control volume are calculated using **Eq. 1**, which is defined as the value of hydraulic resistance from the Hagen-Pouisse Equation[6] for a circular cross-section, smooth-walled microfluidic channel,

$$R = \frac{8\mu L}{\pi r^4} \tag{1}$$

where μ is the dynamic viscosity of the fluid (assumed for water at 25°C, 8.9x10^{-4} Pa s), L is the length of the microchannel and r is the hydraulic radius of the microchannel. Afterwards, the total equivalent hydraulic resistance of all fluids exiting the

control volume *via* the outlet microchannels, $R_{out,total}$, is calculated using **Eq. 2**,

$$\frac{1}{R_{out,total}} = \sum_{i=1}^{n}(\frac{1}{R_{out,i}}) \tag{2}$$

which is the hydrodynamic equivalent of the Parallel Resistors equation used to calculate the effective total electrical resistance of resistors in parallel where $R_{out,i}$ represents each of the individual hydraulic resistances of the outlet microchannels (as previously calculated from **Eq. 1**). The total volumetric flow rate into the control volume, $Q_{in,total}$, is then calculated by taking the sum of all volumetric flow rates from the input microchannels into the control volume. Each volumetric flow rate that leaves the control volume *via* each of the individual output microchannels ($Q_{out,i}$) can then be calculated using the Conservation of Momentum equation in **Eq. 3**,

$$Q_{out,i} = (\frac{R_{out,total}}{R_{out,total} + R_{out,i}})Q_{in,total} \tag{3}$$

Then Conservation of Mass equation is then solved using **Eq. 4**,

$$Q_{total}C_{total} = \sum_{i=1}^{n}(Q_iC_i) \tag{4}$$

where Q_i and C_i are defined as the flow rate (as a function of the proceeding flow rates) and concentration (as a function of the proceeding concentrations) of each of the input fluids into the control volume, respectively (*i.e.* $\sum_{i=1}^{n}(Q_{in,i}C_{in,i})$). Once $Q_{total}C_{total}$ is calculated as a function of the proceeding known input values, this quantity can be defined as equivalent to the total mass leaving the control volume *via* the outlet microchannels (*i.e.* $\sum_{i=1}^{n}(Q_{out,i}C_{out,i})$), and **Eq. 4** is used once more to solve for the flow rate (as a function of the input flow rates) and concentration (as a function of the input concentrations) of each of the output fluids leaving the control volume. Finally, following this convention throughout the 3D microchannel nodal network, the flow rate and concentration of each of the fluids exiting the microchannel network *via* the device outlet microchannels can then be mathematically defined in terms of the initial input flow rates (Q_1, Q_2 in units of μL/min) and concentrations (C_1, C_2 in units of μg/L) of each of the input fluidic species, and subsequently analytically calculated in order to quantify each of the values.

1.6 Commentary on the output flow rate from the prototype device

The analytically derived design equations featured in Figure S2 demonstrate that the fluid flow rate of the fluidic outputs from each device outlet are not identical, as the fluidic resistance on the outer branches of the microchannel network is lower than on the inner branches. As illustrated in Figure S2h, which presents the analytical calculations for the output flow rates as a fraction of the overall input flow rate (identical for each input species to the flow rate input into device Inlet #1, directly above Outlet #1), the output flow rates range anywhere from a high of 1/2 to a low of 1/12 of the input flow rate.

For the purposes of generating the intended concentration gradient, as was confirmed from the theoretical simulations and the experimental fluorescent dye validation, the variable output flow rates are inherent element of the fluid flow characteristics of the device and is not of concern. A practical consequence of the output flow rate variation, however, as was observed during the experimental operation of the fabricated prototype device, the variable output flow rates required the device to be operated at roughly 1 mL/min for upwards of one minute in order to generate sufficient fluidic pressure as to overcome the higher fluidic resistances of the inner microchannel branches and entirely fill the output segments of tubing attached to said device outlets. As a result, waste fluid was generated from the device outlets to the lowest fluidic resistance microchannel network branches during operation of the device. Moving forward, using Hagen Poiseuille hydrodynamic resistance law and branched gradient generator design rules, the inner diameter of the hollow channels can be modified to increase or decrease fluidic resistance of microchannels throughout the network in order to generate equivalent fluid flow rates at each branch of the microchannel network, thereby producing different quality concentration gradients with more equitable output flow rates for more practical experimental utility.

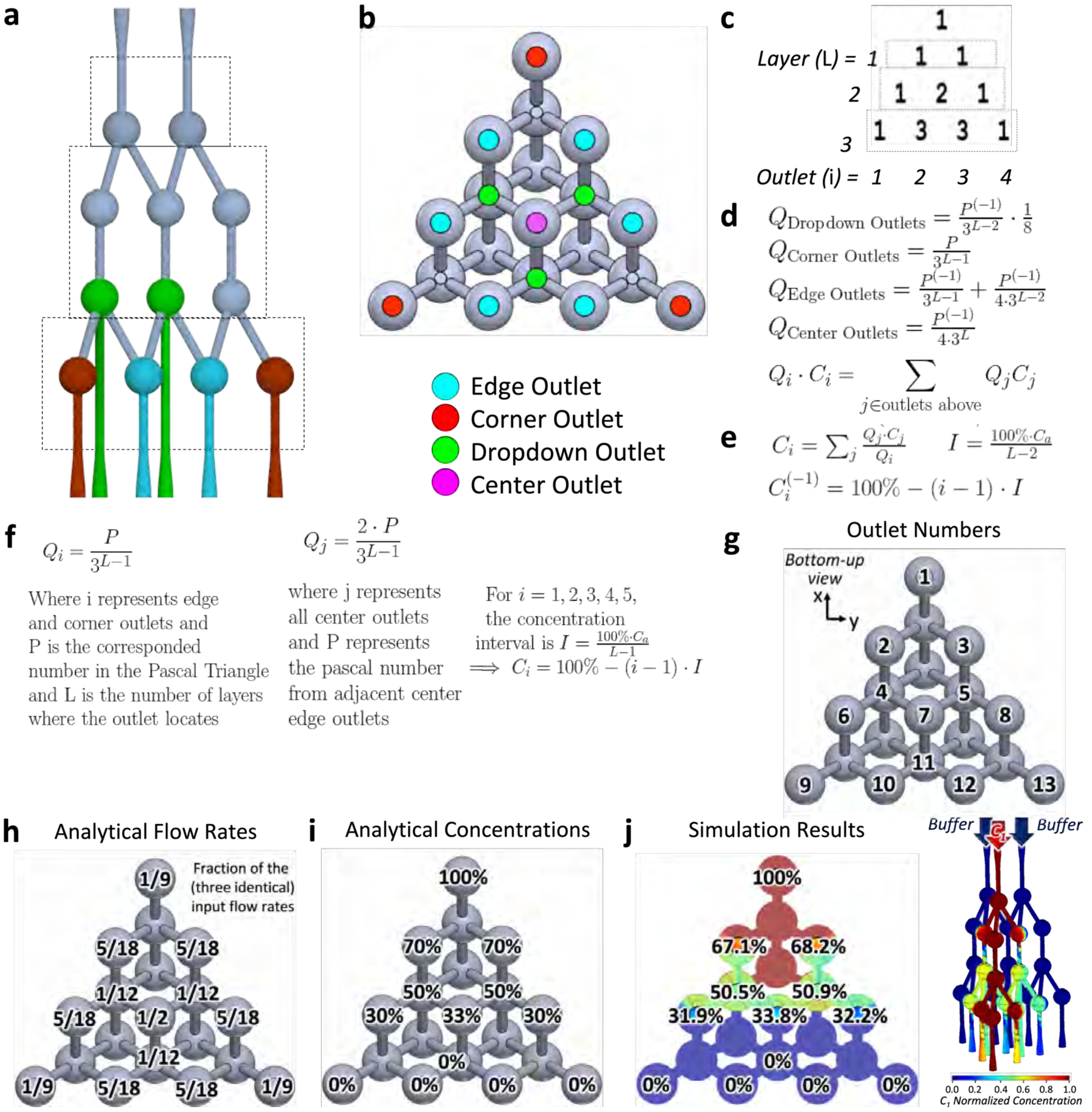

Figure 2. Diagrams, analytical design and equations used in the nodal analysis of the 3D μ-CGG fluidic network. **(a-b)** Microchannel network and outlets labels. **(c)** Pascal's triangle representing the labeling of the outlets on each side of the microchannel network. **(d)** Analytical flow rate out of each device outlet. **(e)** Conservation of mass equations. **(f)** Derived design equations. **(g)** Output numbering convention. **(h)** Analytical calculations for the output flow rate at each device outlet as a fraction of the overall input flow rate, identical for each input species. **(i)** Analytical calculations for the concentration of a single fluidic species (input into device Inlet #1, directly above Outlet #1) at each outlet of the device. **(j)** Theoretical COMSOL simulation results for the concentration of a single fluidic species input into device Inlet #1(*left*) at each outlet of the device and (*right*) visualization of the COMSOL results with input flow rates of 1000 μL/min, simulating the species of interest (*red*) input into Inlet #1 and buffers input into Inlets #2 & #3 (*blue*).

2 Fabrication via Multi-jet 3D printing

The Multi-jet 3D printing approach was determined to be the most suitable manufacturing approach to successfully fabricate the featured devices more thoroughly than other SLA or FDM-based 3D printing equipment, namely: Multi-jet 3D printing has sufficiently high resolution (a limit of no higher than 100 μm; FDM and SLA did not reliably print with this fine of resolution) in order to fabricate the intricate 3D structures enclosed inside microchannels; employs multi-material 3D printing (FDM and SLA 3D printers present only single-material printing capability), specifically prints using sacrificial support material in order to successfully fabricate long enclosed 3D microchannels as well as the complex 3D arrangement of microchannels suspended in 3D space. The Multi-jet 3D printing equipment used in this work is the Projet 3000UHD 3D printer (*3D Systems, SC, USA*), available from the Center for Interdisciplinary Biological Inspiration in Education and Research (CiBER) laboratory, Department of Integrated Biology, Valley Life Sciences Building, UC Berkeley, as well as the Center for Information Technology Research in the Interest of Society (CITRIS) Invention Lab, Sutardja Dai Hall, UC Berkeley.

The Multi-jet 3D printing approach entails layer-by-layer inkjet-like deposition of micro-droplets of photocurable materials roughly 32μm in diameter onto an aluminum build plate[7]. Once each layer of materials is deposited in the X-Y horizontal plane, a UV light source flash cures the materials so that they cure, harden to their final mechanical properties and cool sufficiently such that the build plate can be lowered in the Z direction and the subsequent layer deposited on top of the previous layer. The 3D printing materials have a gel-like solid form when contained inside the commercially-available material cartridges, and are heated inside the deposition head in order to lower the viscosity sufficiently for deposition. The build volume of 127mm x 178mm x 152mm (X x Y x Z directions) and speed of the deposition process enable the simultaneous fabrication of multiple individual microfluidic devices, depending on the size and type of devices being fabricated in roughly four to six hours. It was also experimentally observed that the predominant factors determining total fabrication time were experimentally found to be the overall height of the devices (as it was determined that the printer deposits features faster in the X-Y horizontal plane than in the Z direction; orientation of a given design on the build plate therefore potentially has a significant effect on fabrication time) and the packing density of the devices on the build plate (a more compact arrangement of devices results in a shorter transition time for the print heads to traverse the area in the horizontal plane).

A custom 3D printing software provided by *3D Systems* is used to interface with the 3D printer. Briefly, the 3D printable 3D CAD model in Solidworks (file extension .SLDPRT or .SLDASM) is first meshed by the software, creating a stereolithography file (extension .STL), which is a universally-compatible file for software developed to interface with different fabrication technologies, such as various forms of additive manufacturing systems and CNC milling tools[8]. The .STL file is then uploaded into the 3D printing software, which displays a physical surface of the model and allows the user to position it anywhere on the build plate (default position is contacting the build plate, however the user can also choose to fabricate the devices elevated off the build surface). Multiple .STL files can be added to the digital build plate area in one build. The user can typically also select user-defined print settings such as build quality (higher qualities entail slower deposition times, likely to ensure that the deposited material has the highest accuracy in the X-Y horizontal plane and smoother textured horizontal surfaces), build time (sacrifices build quality) and layer height resolution (more layers printed results in a higher in the Z direction accuracy and smoother textured vertical surfaces, but has a significant effect on fabrication time). Additionally, the software for the Projet 3000UHD 3D printer automatically senses which materials are loaded into the printer itself (using RFID-tags on every material cartridge), and so programs appropriate print settings in the software which are optimized for the given material, which presumably are slightly differently calibrated for various materials.

When ready to print, the user sends the command to the software, which automatically calculates where in the model support material is necessary, using a proprietary algorithm. Areas where the software decides support material is necessary is underneath any overhanging structures, inside enclosed features such as cavities and microchannels and on the bottom of each part between the build plate and the structure, forming a roughly 2mm thick raft which can be broken off of the build plate to remove the part without damaging the structure itself. After automatically placing the support material in the build plate model (which is visualized before submitting the print job), some 3D printing software will allow the user to selectively add or remove support material from different areas on the build plate, however the Projet 3000UHD 3D printer software does not offer the user this ability. The software then creates G-code instructions, the coding language used nearly-universally by manufacturing equipment, which is interpreted by the equipment as instructions for depositing each layer of material. Finally, after a clean, dry aluminum build plate is loaded into the printer, the user selects a command on the equipment itself to begin the printing job. Once the fabrication is finished, the build plate is removed from the 3D printer and placed into a -4°C freezer for 10-15 minutes until the support material forming the raft underneath each part contracts sufficiently (the wax-based support material has a larger coefficient of thermal expansion than both the aluminum and structural material) to weaken the attachment of the support material on the build plate, enabling the user to simply lift off the 3D printed parts. The fabricated parts are then ready for post-processing.

2.1 Discussion on the properties of the 3D printed material used

Throughout this work, the structural material used is Visijet M3 crystal (*3D Systems*), which is a urethane-based polymer matte white in appearance, and while the exact composition of this polymer is proprietary [1], basic information is provided in the material data sheet; the polymer consists of approximately 20-40% urethane acrylate oligomers, 15-35% ethoxylated bisphenol A diacrylate and 1.5-3% tri(propyleneglycol) diacrylate which acts as a curing agent[9–11]. This material was chosen because at the time of this work, it is the most readily commercially available and most affordable material offered by and compatible with the Projet 3000UHD 3D printer, which does have the ability to print using different structural material (albeit one type of structural material can be loaded into the equipment at a time). This material, when cured, is mechanically rigid with an elastic modulus given in the material data sheet as 1.159 GPa[11]; however as previous work from our group has demonstrated, the elastic modulus has been experimentally found to lower, roughly 58-116 MPa[9]. The cured polymer has proven sufficiently ductile to produce robust deformable thin-walled mechanical membranes (thickness roughly 200 μm) capable of repeatable deformations simply using manual force applied by a human finger[5,12,13]. In addition to the structural material, the 3D printer also deposits a support material, Visijet S100 (*3D Systems*), the only support material compatible with *Stratasys* 3D printers, which is a hydroxylated wax-based polymer[9,14] which is yellow in appearance once cured and can be melted into liquid-phase at temperatures around 50°C and removed from the structural material comprising the device during the post-processing procedure[5].

The resulting 3D printed devices appear translucent white-clear on the exterior surfaces, and hollow regions filled with the solid support material appear an opaque yellow. A particular advantage of using the Visijet M3 material to fabricate all of the devices in this work is its usefulness towards microfluidic applications, as the material is sufficiently translucent that thin-walled structures allows visualization of fluids inside microchannels. This ability to visualize the internal fluids inside such 3D printed microfluidic devices can permit fluorescence imaging and optical microscopy of the contained fluid through the material, which is a particular advantage for fully enclosed device in applications where such analytical measurement techniques are necessary.

One potential consideration for 3D printed materials in general microfluidic applications is the absorptivity of the material. A highly porous 3D printed material with a high absorptivity towards colored dye and other target molecules could retain molecules on or inside the surface of the material, leading to optical, chemical or biological cross-contamination from experiment-to-experiment. A material with a low absorptivity is therefore desired in most microfluidic applications, particularly those employing 3D printed materials. Over the course of the experiments performed throughout this work, we did not observe any consequential absorption of colored dye or other visible materials onto the surfaces of the 3D printed materials, which likely indicates a low porosity and low absorptivity towards such size and surface charged molecules. Regardless, we employed the following device treatment procedure, which has been experimentally demonstrated to increase the biocompatibility of the VisiJet M3 polymer employed throughout this work, to flush the interior of the microchannels after each experimental run of the fabricated prototype device in order to limit or prevent any possible experiment-to-experiment contamination due to any similar residual molecules on the surface of the 3D printed material inside the microchannels.

One important concern with most 3D printed microfluidic systems developed for biological applications, and specifically taken into consideration in this work, is to ensure that post-processing measures are taken in order to minimize any cytotoxic effects of the 3D printed material, i.e., material characteristics which inhibit cellular growth and reproduction and/or prove lethal to said organism, which could prove detrimental to the ability to successfully culture bacteria inside the fabricated prototype device[15]. Standard materials used to manufacture conventional microfluidic devices designed for cell culture are typically fabricated using materials proven to be biocompatible[16] for most biological applications, such as PDMS, polystyrene (PS) and polymethylmethacrylate (PMMA)[17]. The biocompatibility of many polymers commonly used in additive manufacturing however, proprietary formulations not outwardly-advertised as meeting established biocompatibility standards[18] in particular, has not been definitively proven[19]. Moreover, previous studies have shown that certain Multijet and SLA materials can be cytotoxic to different cellular organisms during cell culture experiments and viability assays[20,21].

Briefly, one of the primary mechanisms behind polymer cytotoxicity is the presence of un-polymerized residual monomers which are are statistically always present after the process of free radical polymerization of photocurable polymers and become trapped in the polymeric matrix at or near the material surface, eventually leaching into contacting aqueous solutions and proving cytotoxic to various cellular organisms contacting the exposed surface of the polymer matrix[22]. Developing various practical methods to reduce the cytotoxicity of commercially-available post-polymerized 3D printed polymers, without significantly

[1] As an aside regarding 3D printer-compatible polymer chemistry, the exact composition of both of the aforementioned materials is proprietary and is not revealed on material data sheets. Maintaining the exact material composition a closely-guarded trade secret is a common practice in the 3D printing industry, as for many if not all of the commercial SLA and Multi-jet companies produce equipment that is most suitable, and in some cases only compatible, with the material which the company itself produces and sells to the end users of the equipment. The proprietary nature of the material compositions, from a business-perspective, allow commercial companies to ensure a steady source of income in terms of material costs from their customers after the equipment is purchased, but also ensures that the equipment can produce the manufacturing specifications (*e.g.* resolution, material finish, material properties, build speed, reliability of the printing mechanisms) promised to the end user, as the equipment can be well-calibrated in the factory to specific materials produced and controlled by the company, ensuring customer satisfaction.

altering other inherent mechanical properties of the material, is therefore one of the primary focuses in current research involving the engineering of 3D printed biomedical devices[23,24]. Conveniently, the specific acrylate-based 3D printed Visijet M3 polymer used to fabricate the prototype in this work has been previously studied in the context of developing practical ethanol-treatment procedures to reduce the cytotoxicity of the post-polymerized material towards the culture of multi-cellular Zebrafish embryos. The results of this recent work showed that washing of the 3D printed material with a 99% aqueous-solution of ethanol, which is known to increase the solubility of residual acrylic monomers in solution, before cell culture resulted in nearly the same survival rate ($\tilde{9}$0% viable organisms at 72 hours) of multi-cellular Zebrafish embryos cultured inside 3D printed enclosed wells as was observed when culturing the same type of organism in a petri dish made from PS. The cytotoxicity of the 3D printed material was therefore shown to be significantly reduced using this ethanol-based material treatment procedure, as compared the same experiments performed using the 3D printed polymer without any ethanol treatment (showing practically 100% mortality at 72 hours).[25]

Therefore in order to reduce potential cytotoxicity of the fabricated 3D printed prototype device in this work in light of the results demonstrated by the aforementioned study[25], an ethanol solution-based treatment was performed as the final step in the device post-processing protocol, whereby a 90% ethanol solution in DI water was used to flush out the fabricated prototype before a final pure water flush. Moreover, during device operation, between each individual experiment using the device, all fluidic reservoirs were filled with a 90% ethanol solution and run through the fluidic network, removing any residue of previous species from inside the device. This procedure was used to prevent any consequential cross-contamination from any potentially absorbed or residual cytotoxic molecules.

3 Support material removal via mineral oil-based post-processing

One of the most significant advantages of Multi-jet modeling over other 3D printing technologies is the multi-material printing capability which enables the use of a sacrificial support material. This support material is used to produce a physical foundation akin to a scaffold upon which complex geometries enclosed inside cavities or channels, as well as large hollow volumes, can be reliably fabricated without material warpage during fabrication, which would occur without the use of a support material. The mechanical properties of support materials vary fairly significantly, however, from wax-based to hydrogel-based materials[26]. Therefore different post-processing techniques must be developed and applied to successfully and completely remove the different formulations of support materials from inside and around 3D printed components. The advantage of the wax-based support material used in this work is its ability to be easily melted into a liquid state. Our research group developed a custom protocol to remove the sacrificial support material from both the exterior and the interior of the 3D printed components[5], which has been extensively demonstrated in our group's previous work[2,2,9,12,13,27,28]. Briefly, this process entails heating 2 250mL beakers containing commercially-available food-grade mineral or vegetable oil (*e.g.* Bayes High-Performance Food Grade Mineral Oil Wood & Bamboo Conditioner) to roughly 50°C, as the support material is found to be soluble in many tested brands of food grade oils around this temperature[9]. Separate beakers of soapy water (roughly 10:1 water-soap mixture) and clean water are also heated up, concurrently. While the beakers are heating up, the 3D printed components are placed into an oven pre-heated to 75°C for 15-20 minutes, until all of the exterior support material is melted and removed from the exterior, especially the bottom surface, of each 3D printed part. Placing the 3D printed parts on top of a tray lined with 4-5 sheets of standard paper towel assists in wicking away the melted wax from the exterior of the devices, expediting the process, as the support material melts into a liquid-phase at roughly 50°C. The 3D printed components are then transferred to the heated beaker and submerged in the mineral oil, which within 2-5 minutes liquifies the remaining support material on the interior of the 3D printed devices, turning the interior of the devices (which can be visualized through the thin-walled material) translucent yellow in color.

At this point, the wax-mineral oil solution can then be flushed out from the interior of hollow channels. All 3D printed components are designed with a custom-shaped 550μm inner diameter microchannel inlet interface, designed and fabricated onto the end of every exposed microchannel on each device, which facilitates support material removal[5] and universal interfacing with a 20 gauge stainless steel interconnecting couple (model SC20/15, *Instech*, USA outer dimeter 910μm, length 15 mm). In order to physically force the wax solution out of the enclosed regions of the devices, a 10mL hand-held syringe (*Cole-Palmer*) which is attached to a 20-gauge Luer stub (model LS20, *Instech*, needle length 12mm) connected to a length of Tygon microbore tubing (model #06420-03, *Cole-Palmer*, IL, USA) with the other end connected to the steel couple, is filled with the heated oil. The couple at the end of the tubing is connected to the inlet of the 3D printed device microchannel and steady pressure is applied by and the syringe (up to 4 ATM[9] to maintain a physical, leak-free seal between the 3D printed inlet and the couple). This pressurized flow of oil dissolves the wax to form a heated wax-oil solution, which is subsequently flushed out of the inlet on the opposite end of the microchannel. This process is repeated up to three times until the oil inside the 3D printed device is no more yellow in color than the surround oil bath, indicating the wax is fully removed. Afterwards, to ensure that no wax remains inside of the device, clean heated mineral oil from the appropriate beaker is flushed through the device up to three times. In order to remove all remaining oil, heated soapy water from the appropriate beaker is flushed through the device up to

three times, followed by a final flush with heated clean water. At this point, the 3D printed device is allowed to cool back to room temperature and the post-processing procedure is complete.

On the potential effects of the heating up of the structural polymer throughout the post-processing procedure, the glass transition temperature of the structural polymer is listed in the material data sheet as 52.5°C[11], so the temperature of the oil is kept within 55-60°C as to preserve the 3D printed structures. Above this temperature range, the structural material becomes opaque white in color, slightly more ductile and rougher in texture, although when allowed to cool back to room temperature the material retains its original appearance and mechanical properties. Furthermore, experiments have shown that above roughly 80°C, especially when comprising thin-walled membranes and other delicate thin features, permanent plastic deformation and physical damage may occur due to compression, forcing liquid through the device at high manual pressures and poking with sharp tweezers. Therefore by keeping the temperature of the liquids below 60°C at all times, the integrity of the 3D printed components is preserved.

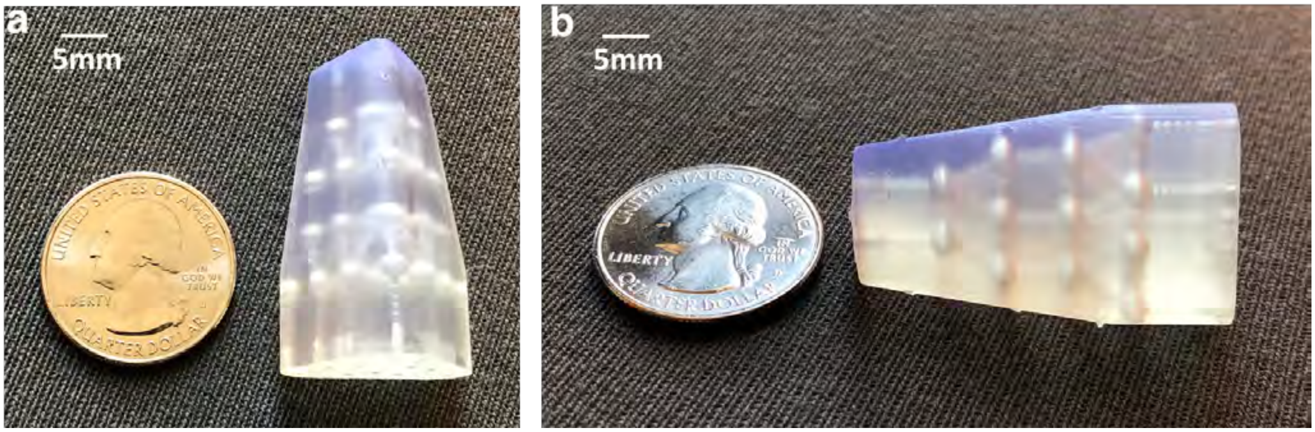

Figure 3. Additional images of the fabricated 3D μ-CGG prototype following post-processing with a US quarter for scale.

4 Further examination of fabricated internal microstructures

In order to further examine the resolution capabilities of the 3D printing technology employed in this work, additional experimental investigations were performed in order to visualize the internal fabricated microstructures comprising entirely 3D printed, fabricated prototype constructs. 3D printed constructs comprising hollow linear smooth-walled microchannels and hollow linear microchannels comprising 3D rifled micromixing structures were 3D printed, with the axial length parallel to the 3D printer build plate, then post-processed to remove all residual support material following the protocols discussed in Sections S2,3. The post-processed constructs were then physically bisected using a sharp razor blade both parallel and perpendicular to the axial length of the microchannels, revealing the exposed 3D rifling and inner surface resolution, and circular diameter with integrated 3D rifling, respectively. Scanning electron microscopy (FEI Quanta 3D FEG FIB, *Thermo Fisher Scientific, MA*) was then used to image the exposed surfaces along the physical bisection in order to observe and make qualitative observation and quantitative measurements of surface roughness and the resolution of physical geometries. Furthermore, 3D laser scanning microscopy (VK-X1000, *Keyence Corporation, IL*) was also employed in order to create a 3D profile map of the exposed surface of the linear microchannel with integrated 3D rifling and to measure feature dimensions. The experimental images from these investigations, along with the results of experimental analysis of the images, are presented in Figure S4a-m.

The surface roughness of the fabricated parts is a factor of the material resolution capabilities of the ProJet 3D printer. This specific 3D printing process entails layer-by-layer inkjet-like deposition of micro-droplets of photocurable polymers, limited in size to $\sim$32μm in diameter by the nozzle diameter of the deposition equipment head[7]. The calibration of the 3D printer deposition head is the other predominant factor determining the feature resolution of the 3D printer, which given proper equipment calibration has previously been shown to result in $\sim$50μm accuracy in lateral feature resolution[9]. The scanning electron images in Figure S4a,c show the resolution of the smooth-walled 3D printed hollow linear microchannel with a designed inner dimension of 750μm. Figure S4a presents a view of the relatively smooth hollow inner channel wall parallel to the axial dimension with no significant intra-channel interrupting geometries, although a precise measurement of diameter from this perspective is difficult to ascertain due to the non-planar surface induced by the physical bisection of the fabricated part. The inner diameter of the as-designed smooth-walled linear hollow microchannel is more easily observed from

the view perpendicular to the axial dimension as shown in Figure S4c which reveals a fabricated inner diameter of roughly 752μm. Additionally of note, this perspective shows the irregular layered texture of the sidewall, which was designed as a radial circumference but was fabricated with intra-layer dimensional innaccuracies. As indicated on the images, the average approximate thickness of these layers, as calculated from individual measurements of each of the twenty-two observable layers in the Z direction, is $\sim$15.20μm. Furthermore, the average approximate length of these layers which extends into the channel itself, as calculated from individual measurements of each of the observable layers in the X-Y direction, is $\sim$32.97μm, which is close to the size of the individual deposited photopolymer droplets. Considering the scale of these surface innacuracies and roughness relative to the characteristic dimension of the microchannel itself, i.e., the $\sim$752μm inner diameter, the maximum error of $\sim$32.97μm in X-Y is approximately 4.38% of the overall dimension, which can be considered an acceptable degree of surface roughness and innaccuracy for the purposes of resolving a hollow circular inner diameter microchannel.

Furthermore, the scanning electron images in Figure S4e,g present the resolution of the 3D printed hollow linear microchannel with integrated 3D rifled micromixing structures embedded in the side walls. The perspective shown in Figure S4e visualizes the repeating 3D rifled microstructures along the length of the otherwise relatively smooth-walled hollow inner channel wall parallel to the axial dimension. The surfaces and edges of the 3D rifled structures are qualitatively rough, which is to be expected, as their thickness is comprised of only a few polymer microdroplets arranged diagonally in the X-Y plane, demonstrating an expected degree of feature innaccuracy. While a precise definition and measurement of their effective thickness is difficult to surmise, an approximate quantification of 3D rifling thickness as $\sim$150μm is indicated in the image. The perspective of the channel inner diameter perpendicular to the axial dimension, on the other hand, as shown in Figure S4g, presents a clearer picture of both the resolved inner diameter of the hollow channel, as well as the cross-sectional dimensions of the fabricated 3D rifling microstructures. This images reveals a fabricated inner diameter of roughly 758μm. Additionally of note, this perspective shows the similar irregular layered texture of the sidewall as shown in Figure S4c, but with the addition of a clear view of the 3D rifling structure as fabricated in the Y-Z plane. An approximate definition and measurement of the roughly triangular-shaped 3D rifling geometry presents a fabricated side-length of the theoretical equilateral triangular cross-section $\sim$160μm, which is close to the as-designed $\sim$150μm long triangular side length. The intra-layer dimensional innaccuracies which result in the layering innacuracies in Z as previously analyzed are apparent in the surface resolution of the 3D rifling, but the overall resolution of the geometry results in a geometry which is relatively apparent.

Moreover, the surface resolution of the fabricated microchannel construct consisting of the 3D rifled microstructures, from the perspective of parallel to the axial dimension of the channel, was further analyzed using the 3D laser scanning image as shown in Figure S4i. This image presents a color gradient mapping with blue representing the lowest portions of the fabricated structures and red representing the highest portions, and the repeating geometries representing the 3D rifled microstructures along the axial dimension of the hollow smooth-walled linear microchannel are apparent. Using this particular profilometric measurement technique, the approximate height and width of one portion of the 3D rifling geometry (Figure S4j) are approximately 89μm and 254μm, respectively, demonstrating a slightly more deformed, i.e., shorter and wider, 3D rifled structure from the previously presented image and from the as-designed dimensions, signifying the known innaccuracies in fabrication resolution. The measurements, however, do confirm the apparent geometries representing the as-designed 3D rifling microstructures.

Finally, the overall roughness of the 3D printed material and geometric surfaces was quantified using the 3D laser scanning image as shown in Figure S4l. The magnitude of the average surface roughness abnormality on a flat 3D printed surface was calculated to be approximately $\sim$30.76μm, which is in agreement with the surface innacuracies and roughness values as determined by the aforementioned scanning electron microscopy images and measurements. Considering the scale of this average surface roughness value relative to the characteristic dimensions of the hollow microchannel, i.e., the $\sim$758μm inner diameter, and the 3D rifled geometries, i.e., the $\sim$254μm width, the surface roughness is approximately 4.06% of the overall inner diameter and approximately 12.1% of the width of the 3D rifled geometries, which can be considered practical degrees of surface roughness and innaccuracy for the purposes of resolving hollow circular inner diameter microchannels with embedded 3D rifled microstructures.

5 Theoretical simulations of output flow rate and concentration

Theoretical simulations using COMSOL were performed to visualize qualitatively, as well as determine quantitatively, the mixing quality and output fluidic species concentrations at each of the discrete fluid outlets for 3D μ-CGG device models incorporating: *smooth-walled* vertical channels, integrated 3D *bulbous μ-mixers* and integrated 3D *rifled μ-mixers*. A negative solids model of each of the designs is created in Solidworks, only for use in the COMSOL studies. Each of these negative solids model part files are imported into separate COMSOL files to perform each individual study, after which a physics-controlled mesh with a normal element size is applied to the model. Each of the three horizontal faces on the three protruding inlet microchannel regions on the top of the microchannel network model are selected as input fluid boundary condition surfaces representing the regions where three theoretical fluid inputs are defined with normal velocity to the surface. Each face on the

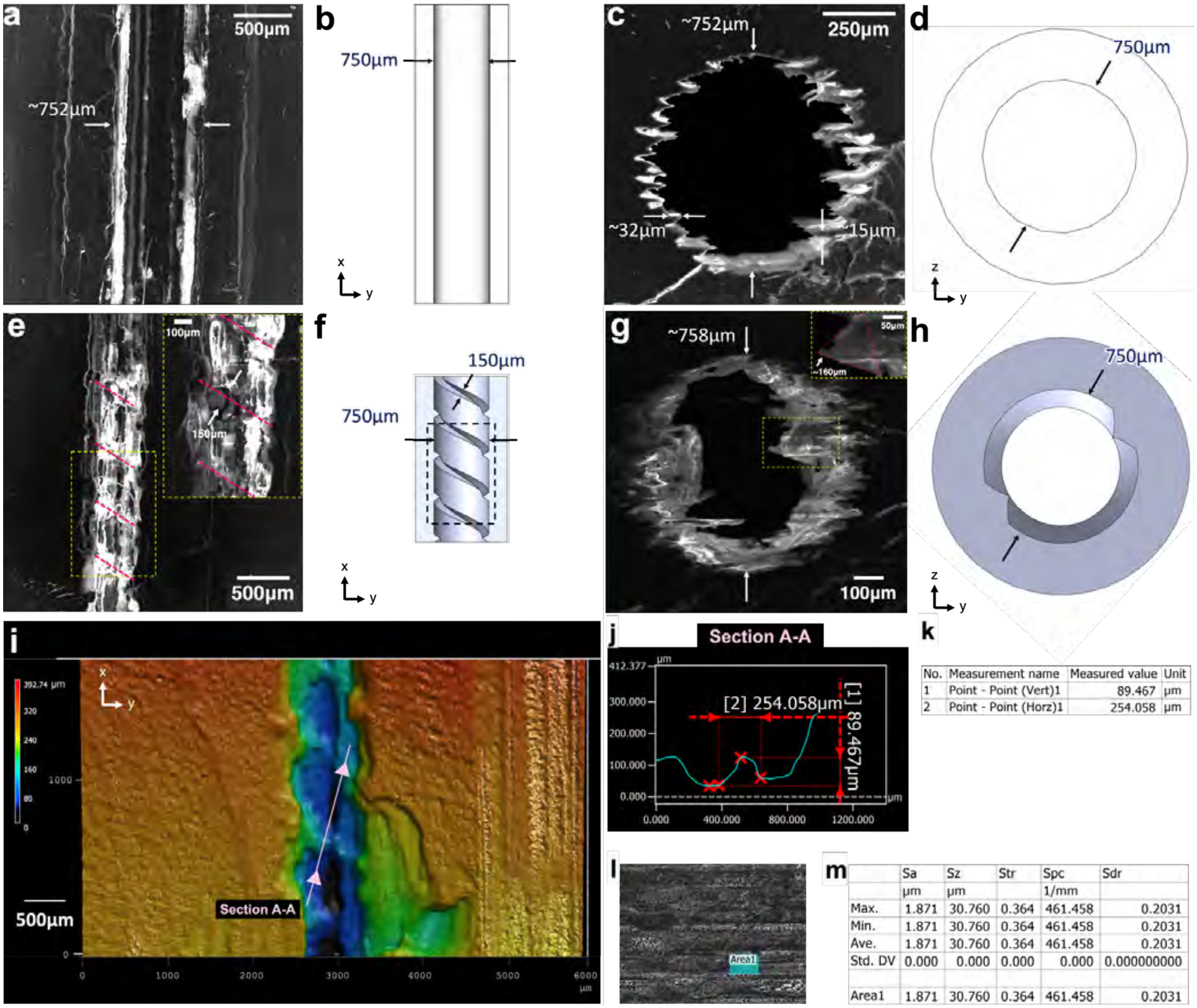

Figure 4. Further investigation of the feature resolution and surface roughness of fabricated internal microstructures from the interior of 3D printed prototype constructs. Scanning electron microscopy (SEM) and 3D laser scanning microscopy (LSM) images with accompanying measurements performed using the equipments' analysis softwares, images taken of fabricated constructs physically bisected using a razor blade to reveal internal geometries. **(a)** SEM image and **(b)** 3D model rendering of a hollow smooth-walled circular diameter microchannel, perspective parallel to the axial microchannel length; perspective perpendicular to the axial microchannel length **(c-d)**, respectively. **(e)** SEM image and **(f)** 3D model rendering of a hollow circular diameter microchannel with sidewall-embedded 3D rifling microstructure, perspective parallel to the axial microchannel length; perspective perpendicular to the axial microchannel length **(g-h)**, respectively. **(i)** LSM image of the same fabricated construct as imaged in (e), Section A-A used to record profilometry measurement of 3D rifling dimensions presented in **(j-k)**. **(l)** LSM image of a flat 3D printed surface revealing surface roughness, used to perform profilometry measurements presented in **(m)** calculating the magnitude of surface roughness.

bottom of the model representing the outlet each microchannel is selected as the only fluid outlet boundary condition surfaces, and all other faces are selected as surfaces for the no-slip solid wall boundary condition. The physics of the study are defined using Eulerian fluid mechanics and approximated using laminar and incompressible fluid flow assumptions to solve built-in fluid hydrodynamic equations. To assign input concentration species, one distinct input species (C_1) is assigned to one of the three fluidic inlet surfaces (referred to as *Inlet #1*), and another input species (C_2) is assigned to both of the two remaining

fluidic inlet surfaces (referred to as *Inlet #1* and *Inlet #2*, respectively). Standard material properties of water as shown in Table 1 were chosen and modeled for the input fluids, and the two input species are defined with the diffusivity constant of fluorescein molecule in water at 25 ° C with initial concentrations $C_{1,o}$ = 1 mol/m^3 and $C_{2,o}$) = 0 mol/m^3.

Variable	Value
ρ	1000 kg/m^3
μ	8.9x10^{-4} Pa s
d_h	750x10^{-6} m
A_d	$\pi\ (\frac{d_h}{2})^2$ m^2
$D_{fluorescein}$	4.25x10^{-6} cm^2 s^{-1}

Table 1. Parameters used in theoretical simulations and experimental data processing for the 3D μ-mixers

Finally, a parameter sweep of discrete input flow rates from 10 to 4000 μL/min as shown in Appendix **Table 2**, was assigned to all three input fluids.

Flow Rate (Q, μL/min)	Reynolds Number (*Re*)	Péclet Number (*Pe*)
10	0.3179	2665.7462
50	1.5896	3328.7308
100	3.1791	6657.4617
150	4.7687	9986.1925
250	7.9478	16643.6542
500	15.8956	33287.3084
1000	31.7912	66574.6167
1500	47.6869	199861.9251
2000	63.5825	133149.2334
2500	79.4781	166436.5418
3000	95.3737	199723.8502
4000	127.165	266298.4669

Table 2. Flow rates and corresponding Reynolds and Peclet Numbers (as calculated using variables in Table 1.1) used as input parameters for theoretical simulations of the 3D *tetrahedral*-based μ-CGG designs.

The ultimate objective each study is to quantify the concentration of C_1 present at every location, especially at each of the outlets of the device. To this end, a unit-less metric of normalized concentration (N) is introduced as defined by Eq. 5,

$$N = \left| \frac{c_1}{c_{1,0}} \right| \tag{5}$$

The effect of the combination, mixing and splitting of each of the three input fluids at each node upstream in the gradient generator microchannel network results in various distributions of C_1 at each outlet, which is reflected by the normalized concentration ranging in magnitude from N = 1 (*i.e.* at that location the concentration of $C_1 = C_{1,o}$) to N = 0 (a complete absence of C_1, *i.e.* $C_1 = 0$). Alternatively, the normalized concentration of C_1 can also be presented in the form of a percentage of $C_{1,o}$ (*e.g. 69% C_1 at a given device output*).

When the simulation is run, the physical equations are solved and the normalized concentration is calculated at every location inside the solids model. The resulting physical distribution of normalized concentration solutions can then be visualized on the outer surfaces (along the inner wall of the actual microchannels), on the outlet faces (on the fluid profiles at the outlets of the actual microchannels) and on discrete profiles perpendicular to the axial fluid flow inside the model.

A critical assumption was made when undertaking the theoretical study of all of the models developed in this work, that due to the symmetry of all of the microfluidic networks designed, the results for the normalized concentration of C_1 when assigned

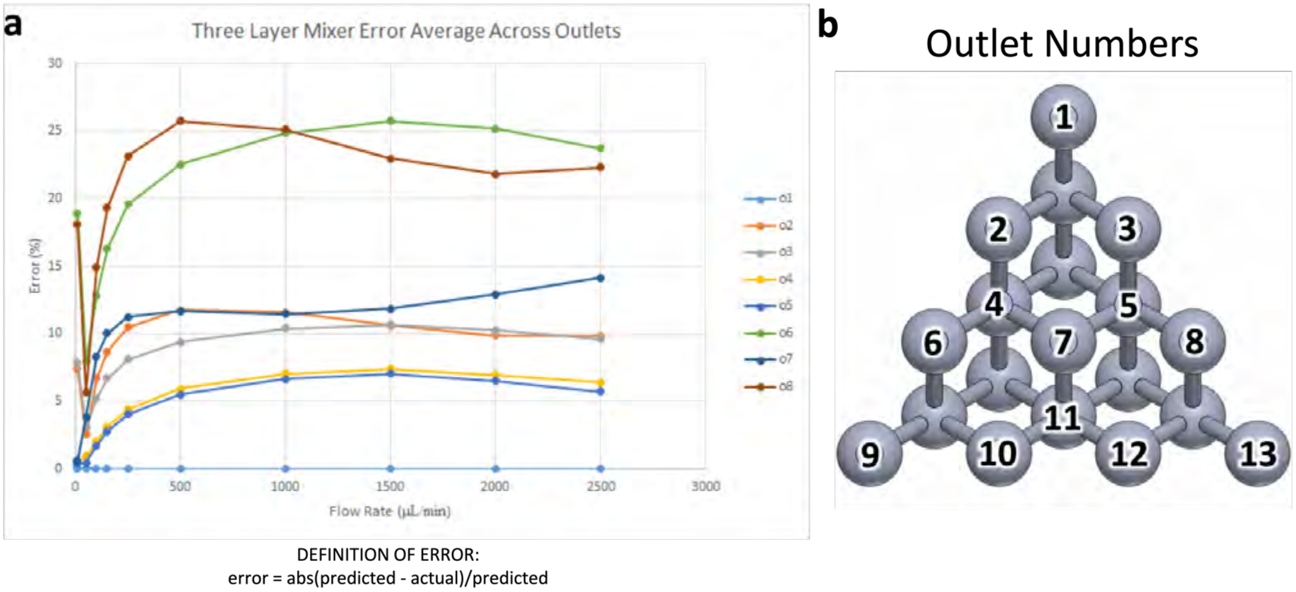

Figure 5. **(a)** COMSOL results for error from the mean value of concentration at device outlets 1-8 (non-zero normalized concentration values) for the simulated output of the 3D μ-CGG design with 3D rifled vertical channels. **(b)** Outlet numbering convention.

to microchannel Inlet #1 will be identical to the results when C_1 is assigned to Inlets #2 & #3. Therefore in this work, only one input species arrangement was assigned and tested, that is C_1 assigned as the input to Inlet #1. In all of these visualizations, a 100% concentration C_1 at any location inside the microchannel network is represented by the color red, 0% concentration C_1 is represented by the color blue and any diluted concentration of C_1 is represented as a color in a gradient between blue and red. The single quantitative value calculated by each simulation is defined as the average normalized concentration value on the entire face of each outlet on each of the microchannels in the model. This represents the average normalized concentration of C_1 in an infinitesimally-small volume of fluid at the outlet of the microchannel of the actual device. Upon completion of each simulation, one normalized concentration value is produced for each flow rate simulated as defined in the parameter sweep, with the average percent error from the mean concentration value of the pixels on the face of each outlet as presented in Figure S5.

6 Experimental materials

6.1 Resazurin metabolic indicator

Resazurin is most frequently available as a sodium salt powder, dark blue in appearance, and serves the function of an oxio-reduction indicator, as the cellular electron receptors that are used in metabolic activity chemically-reduce the molecule to produce the molecule resofurin, which is bright pink in appearance. The rate of reduction of resazurin into resorufin occurs proportionally to the rate of respiration of cells in the medium, therefore the use of resazurin is proven to be a simple, accurate and reproducible method to quantifiably assess the metabolic activity of organisms including animal cells, fungi and bacteria during both aerobic and anaerobic respiration, having been previously demonstrated in numerous cell viability, culture and proliferation assay protocols.[29–32] Additionally, the resazurin oxio-reduction process has been shown to be more sensitive to slighter changes in bacterial density or metabolic conditions than accomplished using conventional optical density-based measurement methods, and therefore is often accepted as a higher-sensitivity method to indicate cellular metabolism than optical analysis in many bacterial viability assays[33].

6.2 Measuring cell density

The two primary laboratory-based protocols conventionally can be used to quantify the density of bacteria in the sample, the agar plate counting (solid culture) method and the spectrophotometric (liquid culture) method. The agar plate counting method is a time-intensive procedure and can only quantify the population density of a given sample after-the-fact, around 24 hours after an initial sample is taken for analysis, but can quantify density with an accuracy as high as roughly 10's of cfu/mL. The spectrophotometric, or OD600, method employs a spectrophotometer to quantify the absorbance of each wavelength of light

transmitted through a liquid culture (known as turbidity) and can provide instantaneous measurements of optical bacterial density (most commonly by measuring the absorptivity of the medium at 600nm, known as an OD600 measurement), but the relationship between turbidity and cell density is only accurately correlated for cell suspensions of around 10^7 cfu/mL and greater.[34] The method used throughout this work to quantify the initial density of the overnight culture of liquid bacteria stock is the spectrophotometric method. A Vernier UV-VIS Spectrophotometer (*Vernier*, OR, USA) is used to take the OD600 measurement of ~2 mL of the medium (spectrophotometer is initally calibrated to an equal volume of sterile LB media), and an online calculator (E. coli Cell Concentration from OD600 Calculator, *Agilent Genomics*, CA, USA) is used to correlate the raw OD600 measurement from the spectrophotometer (unitl-less value) to a relevant cell density value for *E. coli* in cfu/mL. Once the initial cell density of the overnight culture is quantified, to create the required bacterial solution with a density of around $5*10^5$ cfu/mL, a serial dilution of the high concentration media is performed. Protocols used in this work to measure cell density using the spectrophotometric method and for the serial dilution of the liquid cell culture media to prepare the appropriate starting concentration of bacteria stock solution for each experiment can be found in Refs.[34,35], respectively.

7 Experimental methods

7.1 Device operation and experimental collection of generated gradient

All of the constituent fluidic elements in the experimental setup shown in Figure S6 were interconnected using Tygon tubing (*Cole-Palmer*) and steel couples (*Instech*), and all experiments were performed at ambient temperature of roughly 20-25°C. The fabricated 3D μ-CGG prototype was integrated into hardware designed to physically hold the device fixed in place so that the inlet tubing, the microchannel network, and the outlet tubing is maintained in a vertical orientation, shown in Figure S6. The fluid outputs from the device were collected in discrete segments of tubing. The desired volume of the output solution was determined by cutting the segment of output tubing to the required length. For example, given the diameter of the tubing (~762 μm), in order to collect a 30 μL volume solution, the length of the tubing segment was cut to ~15.2 mm. Each tubing segment was routed to a 3D printed tray which aligned each tube with the center of a single well on a standard 96-well plate (#07-200-720A, *ThermoFisher Scientific*).

The MAESFLO microfluidic pressure controller system (*Fluigent*, Paris, France), which is comprised of both the Microfluidic Flow Control System (MFCS) micro-controller and the FLOWELL microfluidic flow sensor platforms and is programmable using the MAESFLOW-dedicated software (*Fluigent*) installed on the computer at the work station, reduces high air pressure delivered from a building source of pressurized air at roughly 7 ATM and delivers precisely-controlled air pressure to three independent fluid reservoirs. Each reservoir contains one of three distinct fluidic species (*e.g.* illustrated in the figure as three distinct antibiotic species) and is interconnected to one of the three respective inlets to the prototype device. Before running each experiment, FLOWELL microfluidic flow sensors (*Fluigent*) controlled using the Microfluidic Flow Control System (MFCS) by Fluigent were placed in-series with fluidic tubing between the fluid reservoirs and the tubing routing to the device. Air pressure was then generated and delivered to the fluid reservoirs, measured by the flow rate sensors, and the air pressure value adjusted until the measured pressure-driven volumetric flow rate reached 1000 μL/min. This air pressure is slightly different each time in the experiments due to various factors including the orientation of all routing tubing, the length of tubing used and the height of the outlet tubing interface relative to the bench top, therefore the appropriate air pressure value found before running each experiment is fixed for the duration of that experiment. This single operational volumetric flow rate was chosen because it was observed during initial testing of both prototypes that around this value, the fluid flow through the prototypes was sufficient to fill all of the interconnected fluid collection tubing attached to each outlet of the device in approximately 30-60 seconds. Slower input flow rates (down to around 500 μL/min) resulted in longer times needed to fill all of the fluid collection tubing which extended the operational time longer than one minute and was deemed too time-intensive. At even slower flow rates (below 500 μL/min) the fluidic resistance of the fluid collection tubing added sufficient resistance as to stop the flow of fluids out of select outlets, which would negatively impact the flow dynamics within the gradient generation network itself. At higher fluid flow rates (larger than 1000 μL/min), waste volume was produced out of the outlets which experienced the highest output flow rates while the outlets which experienced the lowest output flow rates needed longer time to fill the fluid collection tubing, which was deemed too wasteful. The volumetric flow rate delivered to the device was therefore maintained at 1000 μL/min for one minute to permit the fluids inside the microchannel network to reach steady state conditions. When the forward driving pressure is shut off, a vacuum is generated in the fluidic network, originating inside the fluid reservoirs with the only exposed end of the fluidic network being the outlet of each segment of output tubing.

A crucial operational requirement for both experimental setup designs is the use of some mechanism to function as a form of *on-off valves*, which is used to prevent back-flow throughout the network of tubing and through the device itself, whilst allowing the fluids to drain from each of the outlet tubes into the the fluid collection receptacle. If such a mechanism was not employed, for example if the three inlet tubes were suddenly physically removed from the top of the prototype, the vacuum holding the fluids inside the network is broken and as fluid drains from one segment of tubing it is replaced by fluid farther up from the fluidic network inside the prototype itself, contaminating the purity of the steady-state gradient generator output

samples originally contained in each of the tubes. A 3D printed T-junction, as illustrated in Figure 6, was designed with 13 independent microchannel elements (a linear microchannel with an outlet in the center oriented outwards at 90 °, all inner diameters 750 μm) incorporating an inlet (from tubing routing from each outlet of the device) and an outlet (to the latter segment of tubing leading to the fluid collection receptacle). Steel couples are inserted into all 39 inlets to this component and all segments are sealed using hot glue for water-tightness. During operation, a solid block of 3D printed material with 13 holes designed to be aligned with, and fit tightly around, all of the 13 exposed steel couples is attached to the T-junction, effectively shutting *off* these microchannel outlets and creating effectively-linear microchannels. When forward fluid driving air pressure is shut off, a vacuum in the fluidic setup is created. When the 3D printed block is removed, however, all 13 microchannel elements suddenly gain an additional outlet which is exposed to ambient pressure, which permits all of the fluids to drain from the now-exposed latter segments of tubing into the 96-well plate for fluid collection. Employing this approach results in a total time to collect all fluid outputs from the device once the fluid flow is stopped of roughly 5 seconds. The orientation of the outlet tubing routing to the wells on the 96-well plates is illustrated in the diagram shown in Figure 10d.

As previously mentioned in Section 2.1, a 90% ethanol solution was run through the fluidic network between each experiment, removing any residue of previous species from inside the device. Each fluid collection experiment for a specific combination of input fluidic samples was performed in triplicate and collected on two 96-well plates. Prior to the bacteriological experiments, however, flow verification experiments are performed on each prototype in order to empirically validate the fluid flow characteristics of each of the concentration gradient generation microchannel networks. One solution of rhodamine in DI water and two solutions of pure DI water are used, and a red optical filter is used.

7.2 Flow verification experiments

Despite the geometric symmetry of the solids model of the microchannel network design, the assumption cannot be made that the distribution of one species when input into microchannel Inlet #1 will be identical to that achieved when inputting said species into Inlets #2 & #3. The 3D printing process will introduce some degree of physical structural or material asymmetries inside the physical fabricated prototype (*e.g.* randomized reductions in the hydraulic diameter in random locations in microchannels due to polymer deposition error or material warpage during printing). Since the upper limit of resolution of the 3D printer is stated as on the order of 10's of μm, a conservative estimation of the geometrical inaccuracies can be assumed to be on the order to 50-100 μm, an average of 75 μm, which is roughly 10% of the scale of the as-designed hydraulic diameter of the microchannels, and is roughly 50% of the size of the 3D rifling microstructures used inside the 3D rifled μ-mixer-incorporated channels in the fabricated prototype design. It was therefore surmised that such errors are more than likely to induce even slight asymmetries in the distribution of the three distinct fluidic input species throughout the microchannel network. Thus, three separate flow verification experiments were performed on the 3D μ-CGG prototype device in order to analyze the distribution of each of the three inlet fluids separately. To this end, one solution of rhodamine in DI water and two solutions of pure DI water are used; each of the three separate experiment entailed using the rhodamine as the fluidic input to one of the microchannel inlets and DI water as the input to the other inlets, such that separate rhodamine distributions were collected when using rhodamine as the input to microchannel Inlets #1, #2 & #3.

To isolate the fluorescent imaging experiments from sources of background light interference and to achieve the highest contrast fluorescence images as possible, a comprehensive light-isolation fluorescence imaging setup was fabricated. The main component, the light-isolation box, shown in Figure S6(8), consists of a plastic container with the lid attached and placed bottom-up on a bench top, with a hole cut into the side (the panel re-attached using hinges to form an access door) and black felt material lining every interior surfaced (attached using glue). The result is an interior space for which all sources of external light are blocked, and internal light reflection is minimized by the use of the light-absorbent felt. A hole is drilled into the top of the box and a custom-cut rubber PVC pipe fitting used to securely mount a 10 megapixel DSLR digital camera (Canon EOS 1000D, *Canon*, Tokyo, Japan) facing downwards with the lens inserted vertically into the interior of the box. Two additional holes are drilled into the side of the box and used to mount two UV light bulbs which serve as fluorescent excitation light sources. Since the peak fluorescence emission energy of resorufin is $\sim$584 nm, a 585 nm optical filter[36] (*Omega Optical*, VT, USA) is placed in front of of the optical lens of the camera. The filter serves to best isolate any interference caused by the fluorescence of resazurin, the fluid receptacle itself and background light noise. A custom-designed optical filter holder was designed, 3D printed and attached to the front of the camera lens, used to suspend the inserted optical filters in front of the lens itself, covering the full view of the camera. Finally, the lens focus is manually-adjusted every time that a fluid receptacle is placed into the box, and the image settings are manually-adjusted to create optimum brightness and contrast in the images (camera aperture f-stop of 1/5.6, ISO 800 and exposure of 0.25 seconds.

All experimental fluorescence images are analyzed using Fiji image processing software. For each experiment, one image is taken with the center of each well aligned with alignment mark on the center of the camera eyepiece. The fluid receptacle is manually re-positioned for each image. The number of the well (position on the fluid collection receptacle) for each image and the image file name (as read off of the camera display) are both recorded in a lab notebook.

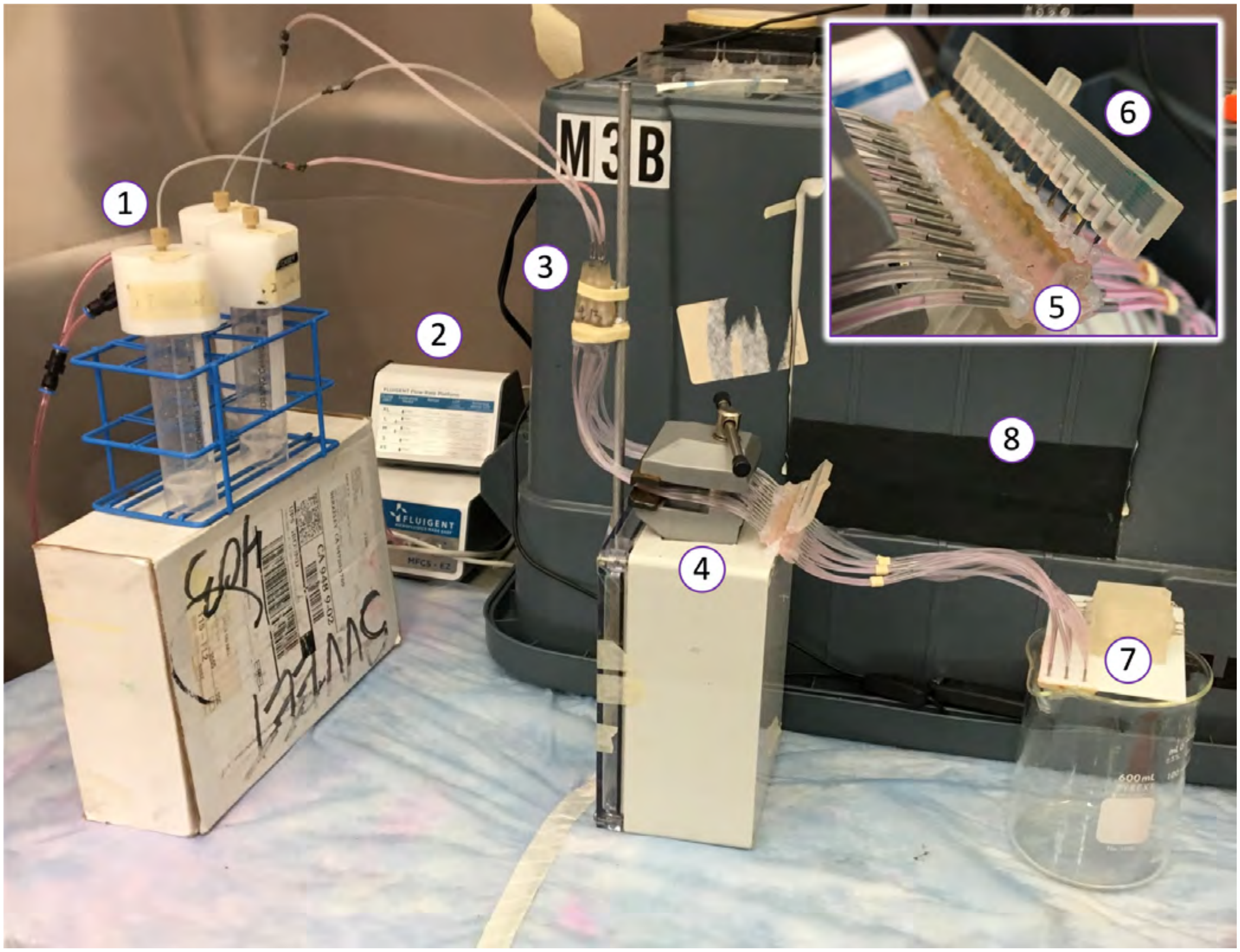

Figure 6. Experimental setup for operation of the fabricated 3D μ-CGG prototype and collection of the generated gradient. **(1)** Independent fluid reservoirs, connected to the **(2)** Fluigent microfluidic controller unit via pink tubing; output tubing connected to the three fluidic inlets on the **(3)** fabricated prototype, mounted vertically. 13 discrete output tubes connected to a **(4)** shut-off mechanism, employing a vice to the pinch tubes. (*Inset*) Tubes are connected to a **(5)** 3D printed T-junction with an attached **(6)** 3D printed block which, when removed, opens the tube to ambient pressure, permitting fluid volume stored downstream in the tube to drain into wells on a 96 well plate at the **(7)** well plate interface. **(8)** The custom-fabricated light isolation box for fluorescent imaging.

7.2.1 Flow verification experimental fluorescent image analysis protocol

The sets of images analyzed from the 3D μ-CGG design experiments are as follows:

- Rhodamine into device Inlets #1 (DI water into all other inlets)
- Rhodamine into device Inlet #2 (DI water into all other inlets)
- Rhodamine into device Inlet #3 (DI water into all other inlets)
- Separate well containing a 30 μL aliquot of pure DI water (0% control well)
- Separate well containing a 30 μL aliquot of pure rhodamine solution (100% control well)

Briefly, for each image using Fiji: (*i*) a circle is drawn to fully enclose the fluid sample well in the center of the image, (*ii*) the red RGB stack channel (best enhancing the contrast of the fluorescence intensity of the emission from the florophore from the background) is selected, and (*iii*) the average pixel intensity inside the circular area is calculated and stored as raw data.

Extracting raw data using Fiji from each individual image from a given set of experimental images:

1. Open Fiji software.
2. Process → Batch → Macro.
3. Select as Input the location of the raw image to be analyzed.
4. Select as Output the location where the processed image is to be saved.
5. Select as Output Format as .TIFF
6. In the coding block, if analyzing **fluorescein** fluorescent images, type the following code:
```
title = getTitle();
index = lastIndexOf(title,”.”);
out_path = path + substring(title,0,index) + ”_avg.csv”;
run(”RemoveOverlay”);
selectWindow(title);
makeOval(2040,700,380,380);
drawOval(2040,700,380,380);
roiManager(”add”);
run(”SplitChannels”);
selectWindow(title + ”(green)”);
roiManager(”select”,0);
run(”SetMeasurements...”,”areamean”);
run(”Measure”);
```
7. In the coding block, if analyzing **rhodamine** fluorescent images, type the following code:
```
title = getTitle();
index = lastIndexOf(title,”.”);
out_path = path + substring(title,0,index) + ”_avg.csv”;
run(”RemoveOverlay”);
selectWindow(title);
makeOval(2040,700,380,380);
drawOval(2040,700,380,380);
roiManager(”add”);
run(”SplitChannels”);
selectWindow(title + ”(red)”);
roiManager(”select”,0);
run(”SetMeasurements...”,”areamean”);
run(”Measure”);
```
8. Click Test. A new image is displays that shows a yellow circle drawn in the location and of the size programmed in the above code, which should be very close to the well in the center of each image. Adjust these values until the yellow circle fits perfectly centered to each well and fits completely inside the wall of the well.
9. When the yellow circle appropriately fits the well, hit Process. This action takes the green (for fluorescein) or red (for rhodamine) RGB stack channel (best enhancing the contrast of the fluorescence intensity of the emission from the respective florophore from the background), measures the average pixel intensity inside the circular area and saves the data in a .csv text file.
10. Open the .csv text file and copy and paste the data into the Excel document, labeling the name of the file, thereby the location of the fluid sample well on the fluid receptacle.
11. Repeat this action for every experimental image. Once finished, the data in the Excel document represents the magnitude of fluorescence intensity in each well.

See Figure S9 for visualized examples.

The metric used to quantify the amount of florophore in each well will be called the Normalized Fluorescent Concentration value (arbitrary units), and is calculated for each device output fluid sample from a given collection of experimental images.

This metric is defined by normalizing the magnitude of fluorescence intensity (amount of fluorescent emission) of a given well containing the fluid sample generated from the gradient generator device and containing a certain percentage of the input florophore concentration, to that present in the 100% control well (maximum concentration of florophore)[29].

Calculating the Normalized Fluorescent Concentration value for each device output fluid sample from a given experimental collection:

1. Open the Excel document representing the experimental data from a given experimental collection
2. The normalized fluorescent concentration value (arbitrary units) is calculated by normalizing the magnitude of fluorescence intensity of a given fluid sample containing the fluid sample generated from the gradient generator device, containing a certain percentage of the input florophore concentration, to that present in the 100% control well, in this case the maximum concentration of florophore[29].
3. Therefore in Excel define in a new region of the spreadsheet the following equation for Mixing Index calculation:
   = ((well of interest) - (0% control well)) / ((100% control well) - (0% control well))
   which defines the Normalized Fluorescent Concentration for a given well of interest as the magnitude of the average pixel intensity from said well, minus the average pixel intensity from the 0% control well representing background fluorescence noise, divided by the magnitude of the average pixel intensity from the 100% control well representing the pure concentration of florophore emission, minus the average pixel intensity from the 0% control well.
4. Apply this equation to all cells representing all fluid receptacle wells.
5. For the data from the experimental tests of the *tetrahedral*-based μ-CGG design, take an average and standard deviation of the normalized fluorescent concentration values previously calculated.
6. These data can now be plotted using Excel.

To calculate this value, the raw data is entered into an Excel workbook and the software used to calculate the Normalized Fluorescent Concentration for a given well of interest as the magnitude of the average pixel intensity from said well, minus the average pixel intensity from the 0% control well representing background fluorescence noise, divided by the magnitude of the average pixel intensity from the 100% control well representing the pure concentration of florophore emission, minus the average pixel intensity from the 0% control well. This calculation is performed on all cells representing the raw fluorescent magnitude values in fluid receptacle wells. For the data from the experimental tests of the *tetrahedral*-based μ-CGG design, an average and standard deviation of the Normalized Fluorescent Concentration values previously calculated are taken. These data can now be plotted.

7.3 Further discussion on the experimental rhodamine concentration distribution results

For the configuration of rhodamine input into Inlet #1 (Figure 5a), the theoretically-predicted concentrations for all outputs are statistically-within the standard deviations of the experimental results (average standard deviation of ∼4.8%). Additionally, the mean normalized concentration values for all outputs are within 10% concentration of the theoretically-predicted values (a maximum of ∼6.0% variation for Well #8, and an average variation of ∼2.0% across all of the wells). Furthermore, for the configuration of rhodamine input into Inlet #2 (Figure 5b), the theoretically-predicted concentrations for all outputs, except for that from Outlet #9, are statistically-within the standard deviations of the experimental results (average standard deviation of ∼3.9%). However, the mean normalized concentration values for all outputs are within 10% concentration of the theoretically-predicted values (a maximum of ∼5.6% variation for Outlet #9, and an average variation of ∼3.0% across all of the wells). Finally, for the configuration of rhodamine input into Inlet #3 (Figure 5c), the theoretically-predicted concentrations for all outputs, except for Outlets #9 & #11, are statistically-within the standard deviations of the experimental results (average standard deviation of ∼3.1%). However, the mean normalized concentration values for all outputs are within 10% concentration of the theoretically-predicted values (a maximum of ∼4.9% variation for Outlet #9, and an average variation of ∼1.7% across all of the outlets).

One probable contributing factor to the observed discrepancy in the mean experimental values and theoretical results could be that the physical morphology of the prototype microchannel network is different enough from the 3D model used in the COMSOL simulation (*e.g.* if residual support material is present at any location inside the microchannel network or if the inner dimensions of the microchannels suffered warpage during fabrication), the hydrodynamics of the fluid flow inside the network could be altered sufficiently as to produce fluid flow patterns different from those simulated resulting in an inaccurate gradient. An additional factor could be that the short lengths of most microchannels and nodal mixing units which simply utilize a hollow mixing bulb (and do not incorporate any mixing enhancement microstructures, for example) result in incomplete fluidic mixing in each node in the gradient generation network; this incomplete mixing would compound the effects of any physical

morphological inaccuracies on the flow dynamics, and resulting in consistently inaccurate overall gradient generation results. In order to evaluate these potential causes further, the experimental accuracy and precision of multiple fabricated prototype devices would need to be evaluated (three prototypes would be required to produce a statistically-satisfactory sample size).

7.4 Bacteriological experiments

Bacterial sensitivity to specific antibiotics varies between strain type and usually evolves over the lifetime of the bacterium[37,38]. As a result, the experimentally-determined MIC values reported in the literature for a given antibiotic compound can vary, often fairly significantly, between different bacterial strains and even study-to-study for the same strain of bacteria[39,40]. Therefore, a range of documented MIC values for each antibiotic are listed in Figure S13. Moreover, an antibiotic concentration double that of an approximate average of reported MIC values from the literature was chosen for the input antibiotic stock concentration such that the "middle" of the generated gradient will represent the approximate MIC value. AST is recommended each time a definitive evaluation of the MIC values for a certain strain of bacteria and antibiotic is desired. Therefore, discrepancies between the experimental MIC values from this work and those documented in the literature are likely due to the variable bacterial strain-dependent antibiotic sensitivity characteristics of the strains used.

A conceptualized cell viability assay for MIC determination using resazurin is illustrated in Figure S7. Here, bacteria and resazurin solutions are each added to two separate wells on the fluid receptacle containing two different concentrations of antibiotic solutions, in one well antibiotic below its MIC value for that specific bacteria (*top*) and in one well antibiotic higher than its MIC value (*bottom*). Upon incubation, the bacteria in the *top* well proliferate in the presence of the sub-MIC value concentration of antibiotic and therefore metabolize the resazurin in the solution, eventually reducing all of the resazurin into resorufin and turning the color of the entire solution visibly pink (due to the color of the resorufin molecule). The proliferation of the bacteria in the *bottom* well is inhibited, however, by the antibiotic, therefore over the same course of time during incubation none of the resazurin is reduced and the well remains visibly blue. This color change is often used as a qualitative *growth-no growth* metric in basic cell viability assays, as can be seen on the *righthand* side of Figure S7 showing an image of an experiment employing a 3D printed fluid receptacle where the four labeled wells contained antibiotic below the MIC value, the rest contained antibiotic above the MIC value.

For all bacteriological experiments, after all of the fluidic outlets from the 3D μ-CGG device (30 μL each) are collected in their corresponding wells, two pre-prepared solutions (in aliquots of 30 μL each) are manually-pipetted into each well: *(i)* a solution of a bacterial metabolic indicator molecule called *resazurin* and *(ii)* an inoculation of ampicillin-resistant *E. coli* bacteria. The duration of incubation of $\sim$100 μL volumes of bacteria solutions in standard micro-broth dilution resazurin protocols typically varies from $\sim$3 hours[29] to $\sim$12 hours[41], and can be as high as 24 hours using conventional macro-broth dilution techniques[42]. In the protocol used in this work, the fluid receptacles are incubated an average of $\sim$5 hours, until a visible gradient of colors is observed, as can be seen in actual experimental images in Figure S9.

Resazurin cell viability protocols are also well suited for quantitative analysis via fluorescent microscopy[32], because whereas resazurin is a weakly-fluorescent molecule, resofurin, which is highly-fluorescent with a peak excitation energy at $\sim$579 nm and peak emission energy at $\sim$584 nm. Therefore for all bacteriological experiments, a strip of green LED lights attached to the ceiling of the box serves as a source of top-down excitation of resorufin in the fluid samples using green light[29]. Since the peak fluorescence emission energy of resorufin is $\sim$584 nm, a 585 nm optical filter[36] (*Omega Optical*, VT, USA) is placed in front of of the optical lens of the camera. The filter serves to best isolate any interference caused by the fluorescence of resazurin, the fluid receptacle itself and background light noise.

7.4.1 Further discussion on each evaluated antibiotic compound

Tetracycline is a naturally-derived antibiotic class which been available as a generic medication since the 1970's and has been one of the safest and effective broad-spectrum medications used to treat serious conditions such as syphilis, cholera, malaria and the plague, but is also commonly-prescribed to treat more benign conditions such as acne. Tetracycline works by interfering with protein synthesis in bacterial cells, and most AST-evaluated bacterial strains, with the exception of *Pseudomonas aeruginosa* (*P. aeruginosa*) and *Proteus mirabilis*, which have demonstrated naturally low-to-intermediate sensitivity towards tetracycline. Tetracycline is also a useful antibiotic in multi-drug treatments used to treat AMR-related infections, such as bacterial peptic ulcers.[43,44]

Ciprofloxacin has been a clinically-available antibiotic since the late 1980's, and is used today to combat UTI's, respiratory infections, gastroenteritis, endocarditis and typhoid fever caused by bacteria such as *E. coli*, *E. faecalis* and *Salmonella enterica*. The working mechanism of ciprofloxacin is by inhibiting the DNA replication and cellular reproduction. Since ciprofloxacin is widely commercially-available and inexpensive to produce, it has historically been used worldwide over the past few decades to treat relatively-minor bacterial infections which would be easily-treatable with other more narrow-spectrum classes of antibiotics; thus, many strains of the aforementioned bacteria have developed low susceptibility, and in some cases very strong resistance, towards ciprofloxacin. In fact, developed-AMR towards ciprofloxacin has been observed even over the course of treatment of a single instance of an infection. The most commonly ciprofloxacin-resistant bacteria are UTI-causing strains of *E.*

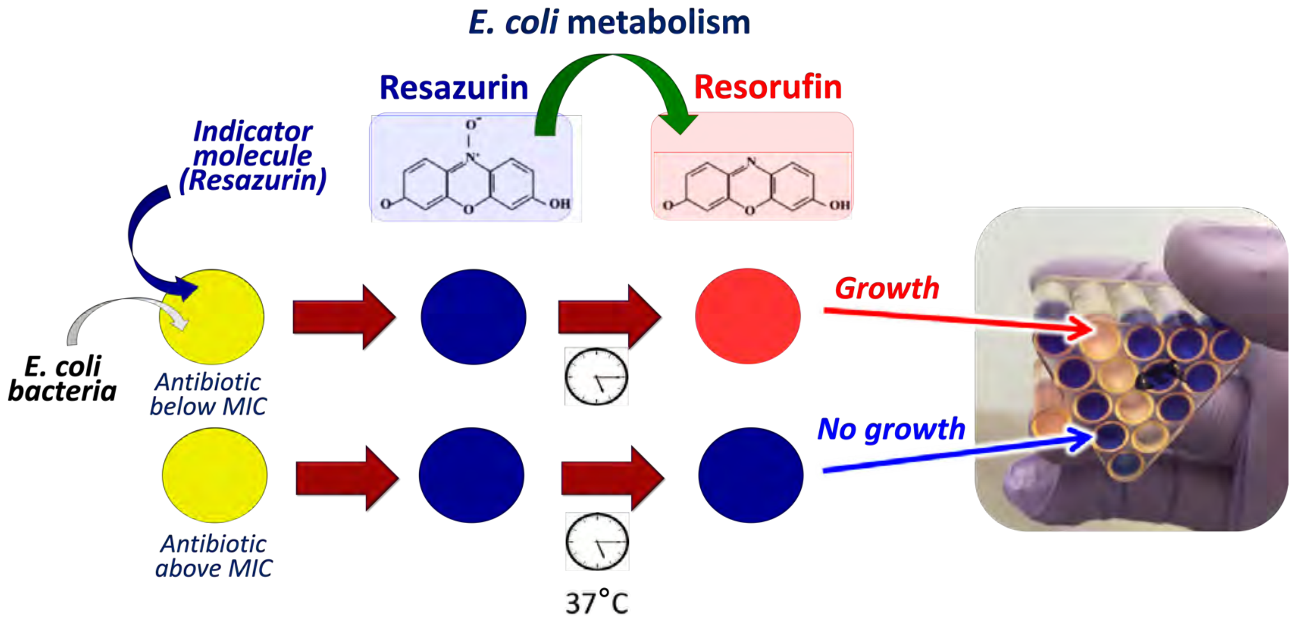

Figure 7. Conceptualized cell viability assay for MIC determination of a specific antibiotic for a specific strain of bacteria, showing the reduction of resazurin into resorufin during incubation and the associated color change of the fluid samples in two wells with two different concentrations of antibiotic solutions: below the MIC value in the *top* well, where uninhibited bacterial proliferation signified by the reduction of resazurin turning the fluid sample from blue to red; and above the MIC value in the *bottom* well, where no resorufin is produced keeping the well blue. Also shown is an image (*far right*) of an actual demonstration experiment employing a 3D printed fluid receptacle where the labeled wells contained antibiotic below the MIC value, the rest contained antibiotic above the MIC value, showing the potential for the resazurin method to be used as a qualitative *growth-no growth* metric of cell viability.

coli, therefore the prescription of ciprofloxacin to treat UTI's is usually precluded by AST evaluation on patient samples used to identify effective MIC values for non-AMR bacterial strains. Ciprofloxacin is often useful in combination with other antibiotics to treat common otherwise AMR-related bacterial infections including UTI's, however, so CDS involving ciprofloxacin is of great clinical importance.[45–48]

Amikacin is generally considered a safe and highly clinically-useful antibiotic first introduced in the 1970's, and is particularly effective in antibiotic combination treatments used to treat otherwise AMR-related bacterial infections such as serious UTI's, tuberculosis and bacterial meningitis caused by bacteria such as *E. coli*, *Enterobacter cloacae*, *S. pneumoniae*, *P. aeruginosa* and *Proteus mirabilis*, by interfering with RNA synthesis. Most bacteria demonstrate susceptibility towards amikacin, mainly when in combination with other classes of antibiotics, but some AMR strains of bacteria such as AMR-related UTI-causing *E. coli* demonstrate intermediate resistance to the antibiotic on its own. As a result, AST evaluation of amikacin, especially CDS of pair-wise combinations including amikacin, is highly-recommended in clinical and drug development settings around the world.[45,49–52]

7.4.2 Protocol For Quantifying Normalized Growth from Experimental Fluorescent Image Analysis

To begin:

1. Create one folder for each set of images from a single well plate tested, which contains: an excel data file (for storage of raw data), all of the raw images (.jpeg files taken from the SD card from the DSLR camera) and the processed images produced by Fiji.

2. Sets of experimental images (one centered over each well on the fluid receptacle) analyzed for the *tetrahedral*-based μ-CGG design:

   - Tetracycline into device Inlet #1 (LB media into all other inlets)
   - Ciprofloxacin into device Inlet #1 (LB media into all other inlets)

- Amikacin into device Inlet #1 (LB media into all other inlets)
- Tetracycline into device Inlet #1, Ciprofloxacin into device Inlet #2
- Amikacin into device Inlet #1, Ciprofloxacin into device Inlet #2
- Tetracycline into device Inlet #1, Amikacin into device Inlet #2
- Amikacin into device Inlet #1, Ciprofloxacin into device Inlet #2, Tetracycline into device Inlet #3
- Separate well containing a 30 μL aliquot of pure LB media (0% control well)
- Separate well containing 30 μL LB media, 30 μL resazurin solution and 30 μL antibiotic solution (100% control well)

Briefly, for each image using Fiji a similar procedure to that previously described is followed, whereby the average pixel intensity on the green stack image of the region defining each well is calculated and stored as raw data. Bacterial proliferation in each well is quantified using the metric of the Normalized Growth value (arbitrary units), and is calculated for each device output fluid sample from a given collection of experimental images by normalizing the magnitude of fluorescence intensity (from the amount of resorufin produced) of a given well containing a certain percentage of the input antibiotic concentration, to that present in the 100% control well, in this case the maximum growth present in the case where no antibiotic is present[29]. To calculate this value, the raw data is imported into an Excel workbook and the software used to calculate the Normalized Growth value for a given well of interest as the magnitude of the average pixel intensity from said well, minus the average pixel intensity from the 0% control well representing background fluorescence noise, divided by the magnitude of the average pixel intensity from the 100% control well representing the maximum concentration of resorufin produced possible, minus the average pixel intensity from the 0% control well. This calculation is performed on all cells representing the raw fluorescent magnitude values in fluid receptacle wells.

Extracting raw data using Fiji from each individual image from a given set of experimental images:

1. Open Fiji software.
2. Process $\rightarrow$ Batch $\rightarrow$ Macro.
3. Select as Input the location of the raw image to be analyzed.
4. Select as Output the location where the processed image is to be saved.
5. Select as Output Format as .TIFF
6. In the coding block, type the following code:
$title = getTitle();$
$index = lastIndexOf(title, "."); $
$out_path = path + substring(title, 0, index) + "_avg.csv";$
$run("RemoveOverlay");$
$selectWindow(title);$
$makeOval(2040, 700, 380, 380);$
$drawOval(2040, 700, 380, 380);$
$roiManager("add");$
$run("SplitChannels");$
$selectWindow(title + "(green)");$
$roiManager("select", 0);$
$run("SetMeasurements...", "areamean");$
$run("Measure");$
7. Click Test. A new image is displays that shows a yellow circle drawn in the location and of the size programmed in the above code, which should be very close to the well in the center of each image. Adjust these values until the yellow circle fits perfectly centered to each well and fits completely inside the wall of the well.
8. When the yellow circle appropriately fits the well, hit Process. This action takes the green RGB stack channel (best enhancing the contrast of the fluorescence intensity of the resorufin emission isolated by the 585 nm filter from the background), measures the average pixel intensity inside the circular area and saves the data in a .csv text file.
9. Open the .csv text file and copy and paste the data into the Excel document, labeling the name of the file, thereby the location of the fluid sample well on the fluid receptacle.

10. Repeat this action for every experimental image. Once finished, the data in the Excel document represents the magnitude of fluorescence intensity in each well.

Calculating the Normalized Growth value for each device output fluid sample from a given experimental collection:

1. Open the Excel document representing the experimental data from a given experimental collection

2. The normalized growth value (arbitrary units) is calculated by normalizing the magnitude of fluorescence intensity of a given fluid sample containing the fluid sample generated from the gradient generator device, containing a certain percentage of the input antibiotic concentration, to that present in the 100% control well, in this case the maximum growth present in the case where no antibiotic is present[29].

3. Therefore in Excel define in a new region of the spreadsheet the following equation for Mixing Index calculation:
= ((well of interest) - (0% control well)) / ((100% control well) - (0% control well))
which defines the normalized growth value for a given well of interest as the magnitude of the average pixel intensity from said well, minus the average pixel intensity from the 0% control well representing background fluorescence noise, divided by the magnitude of the average pixel intensity from the 100% control well representing the maximum concentration of resorufin produced possible, minus the average pixel intensity from the 0% control well.

4. Apply this equation to all cells representing all fluid receptacle wells.

5. For the data from the experimental tests of the *tetrahedral*-based μ-CGG design, take an average and standard deviation of the normalized growth values previously calculated.

6. These data can now be plotted using Excel.

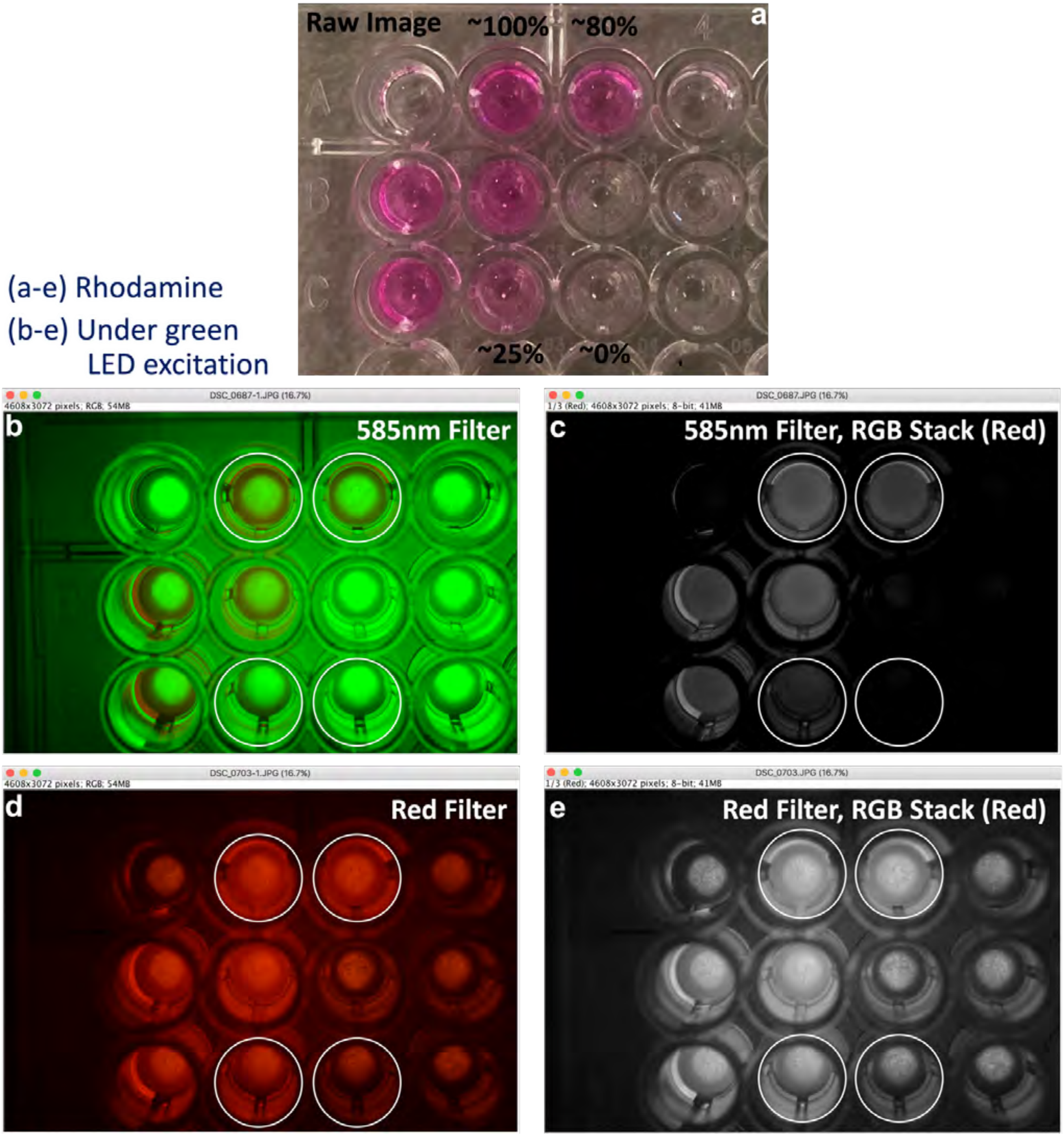

Figure 8. Visual aid showing example images of various stages of Fiji image analysis of a matrix of wells representing a gradient of rhodamine on a 96-well plate. **(a)** raw image of 96-well plate used for analysis; **(b)** raw image of 96-well plate imaged under a 585 nm bandpass filter; **(c)** Fiji-processed image of the red RGB stack channel of this image; **(d)** raw image of 96-well plate imaged under a red bandpass filter; **(e)** Fiji-processed image of the red RGB stack channel of this image. **(b)-(e)** Show four circles overlaid on the images as drawn by Fiji.

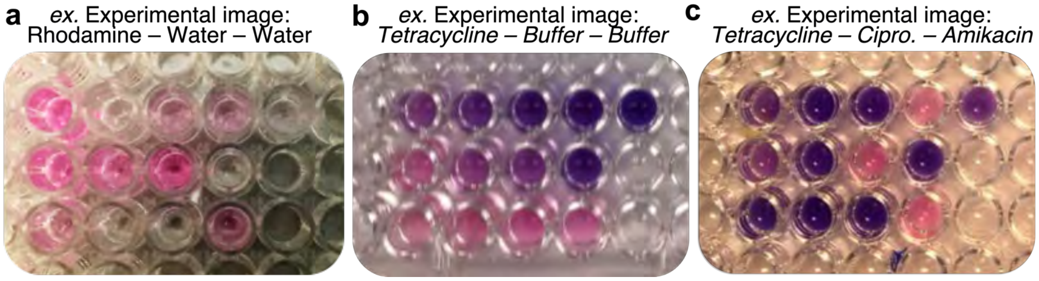

Figure 9. Example images of experimental results from fluid flow verification and bacteriological experiments, fluids collected and analyzed on 96 well plates, images recorded with handheld iPhone camera for visualization purposes only. **(a)** Rhodamine - DI water - DI water gradient. **(b)** Indication of bacterial proliferation, showing resazurin-to-resorufin metabolic reaction in specific well having turned blue-to-pink, after well plate incubation of bacterial stock and growth media in the presence of a gradient of Tetracycline antibiotic - LB media buffer - LB media buffer outputs collected from the device. **(c)** Similar bacteriological experimental results to (c), rather in the presence of a gradient of Tetracycline - LB Ciprofloxacin - Amikacin antibiotics collected from the device.

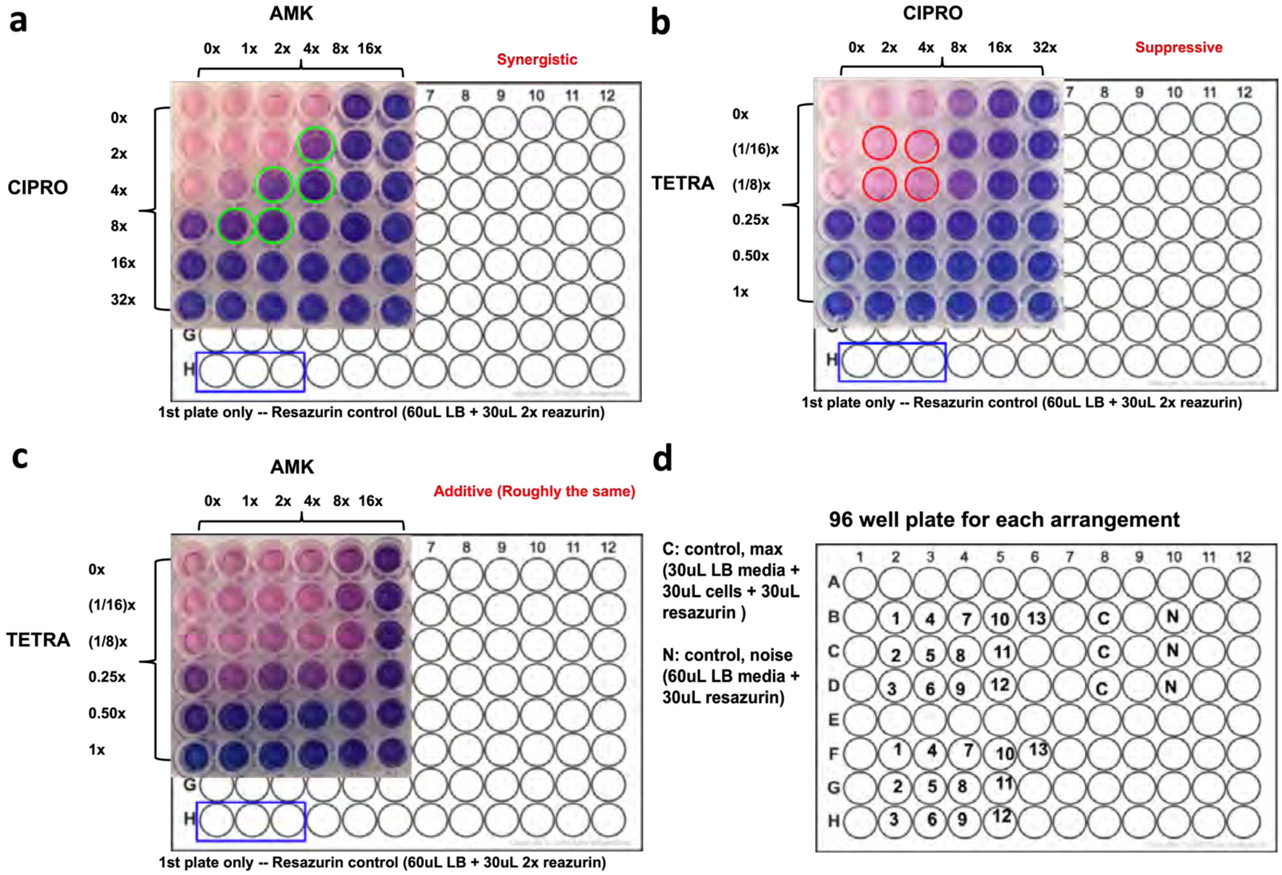

Figure 10. Bulk two-antibiotic combination drug screening experimental results of bacterial growth in manually-pipetted antibiotic solutions. **(a)** Ciprofloxacin and amikacin showing synergistic behavior in the green wells. **(b)** Ciprofloxacin and tetracycline showing antagonistic behavior in the green wells. **(c)** Tetracycline and amikacin showing additive behavior. **(d)** 96 well plate arrangement.

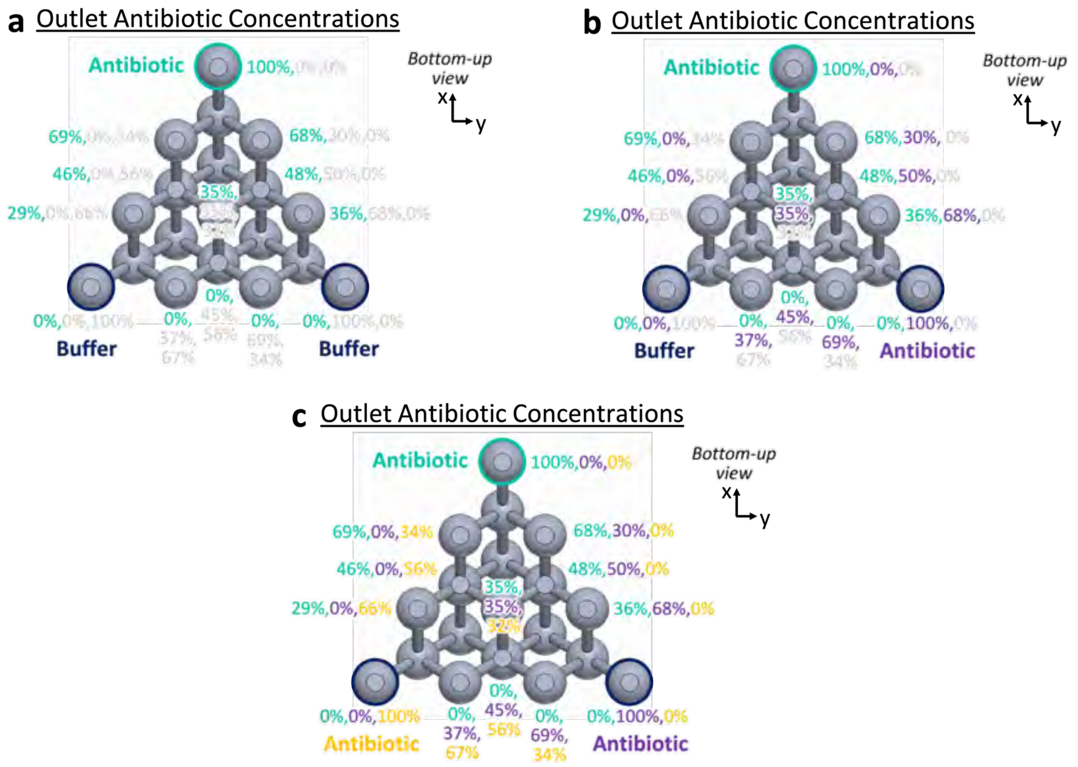

Figure 11. Experimental fluid flow verification results culminating in a quantitative determination of the concentration of each input fluidic species from each inlet at each outlet of the device, given as a percentage of the initial concentration of each input fluidic species. Indication of concentrations for **(a)** single antibiotic MIC experiments (antibiotic-buffer-buffer), **(b)** pair-wise CDS experiments (antibiotic-antibiotic-buffer) and **(c)** three-way CDS experiments (antibiotic-antibiotic-antibiotic).

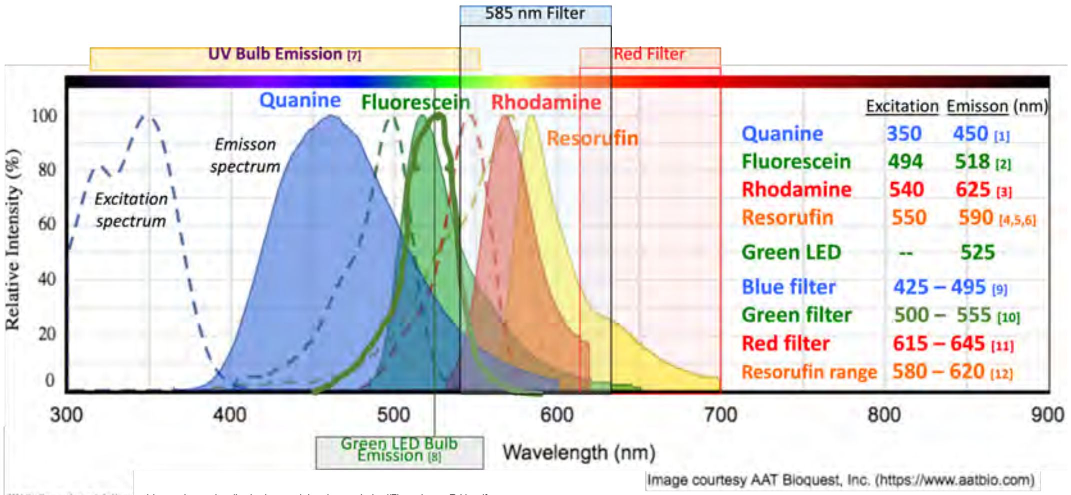

[1] http://www.ohsu.edu/xd/research/research-cores/small-animal-research-imaging-core/upload/Fluorochrome-Table.pdf
[2] http://www.ohsu.edu/xd/research/research-cores/small-animal-research-imaging-core/upload/Fluorochrome-Table.pdf
[3] http://www.colby.edu/chemistry/CH332/laboratory/Quinine%20fluorescence.pdf
[4] http://www.biotech.iastate.edu/facilities/flow/Fluorochromes.pdf
[5] https://www.researchgate.net/figure/260732521_fig2_Figure-1-Excitation-and-emission-spectra-of-resorufin-standard-200pmolmL-1-dissolved
[6] https://www.sigmaaldrich.com/content/dam/sigma-aldrich/docs/Sigma/Product_Information_Sheet/1/r3257pis.pdf
[7] http://www.topbulb.com/specialty-bulbs/ultraviolet
[8] https://www.monstergardens.com/Monster-Super-Green-LED
[9] http://midopt.com/filters/bp470/
[10] http://midopt.com/filters/bp525/
[11] http://midopt.com/filters/bp635/
[12] https://www.promega.com/-/media/files/resources/protocols/technical-bulletins/101/celltiter-blue-cell-viability-assay-protocol.pdf

Figure 12. Visual excitation and emission spectra for all chemicals and optical filters used in this work.

7.5 Antibiotic stock solution and MIC value concentrations in all experiments

Base Molecule / Species	Stock Solution	In 100% Output Well[1]
Amikacin	~48 mg/L	~16 mg/L
Tetracycline	~1.5 mg/L	~0.50 mg/L
Ciprofloxacin	~0.19 mg/L	~0.096 mg/L
Ampicillin	~96 mg/L	~32 mg/L
Resazurin	~125 mg/L	~42 mg/L
K12 Ampicillin-resistant *E. coli*	~3*10^6 cfu/mL	~1*10^6 cfu/mL

Antibiotic	Documented MIC	MIC (Bulk)	MIC (Device)
Ampicillin	~4-8[b,c,d,e] mg/L	N/A	N/A
Amikacin	~16[g] - 20[d] mg/L	~16.00 mg/L	~11 mg/L
Ciprofloxacin	< ~1[a,c,d,e,f] mg/L	~0.064 mg/L	~0.050 mg/L
Tetracycline	~1-2[e,f] mg/L	~0.50 mg/L	~0.26 mg/L

[1]Determined from bulk MIC well plating study experiments; 90μL total

Figure 13. Tabulation of all antibiotic stock solutions and experimentally-determined MIC value concentrations from all experiments. Citations: a[53], b[39], c[40], d[54], e[37], f[38], g[52].

8 Future directions

Moving forward, 3D printing approaches, such as the Multijet technique demonstrated in this work, will enable biomedical researchers to simply modify the digital designs of different classes of 3D μ-CGG devices to tailor the 3D gradient generation performance and number of discrete fluidic inputs and outputs of their CGG biomedical tools. These devices can then be rapidly-prototyped and fabricated in-house using a commercially-available 3D printer, in a clinical point-of-care setting, customizing μ-drug cocktail concentrations to meet the needs of specific experimental applications, *on-demand*, such as for replicating specific chemokine gradient environments and producing specific combinations of sensitive chemotherapeutic drugs to study precisely-dosed multi-drug cancer cell growth inhibition, *on-chip*.

8.1 Further design considerations to increase AST drug concentration dilution range

The 3D μ-CGG design demonstrated in this work features 3 and 13 distinct fluidic inlets and outlets, respectively. As a result, the device is capable of generating a four-fold dilution of three distinct fluidic species, simultaneously. To widen the context of the experimental demonstrated results in this work, the throughput of the fabricated prototype can be compared to those of conventional and state-of-the-art AST approaches. Conventional AST methods via gold standard broth macrodilution methods have been used to generate upwards of 2-3 log serial dilutions[55]. These approaches, however, typically employ highly manual labor intensive and repeatative fluid handling procedures in order to create such numerous dilutions. Robotic pipetting and fluidic deposition systems are therefore often used to perform automatic fluid handling; while effective at reducing manual labor requirements and fluidic handling time as compared to manual processes, these robotic systems are often too expensive and bulky for widespread adoption. Moreover, the macro-volume dilutions which are produced by these approaches are nearly universally used in further microbroth-based analysis on standard 96 well plates and have limited practicality towards further microfluidic-based analysis.

The promise of enabling both on-chip, passive concentration gradient generation generating at least 4-fold dilutions, combined with automated on-chip fluidic analysis in one compact microfluidic system promises advantageous applicability in many laboratory settings. Indeed, state-of-the-art microfluidic-based AST platforms target exactly that objective. Today, conventional microfluidic-based platforms which are capable of generation of discrete concentration gradients routinely accomplish generation of 4-fold[29,56–58], 3-fold[59] and as low as 2-fold drug solution dilutions[60], and enable on-chip micro-volume fluidic AST analysis. These systems, however, as previously discussed, are limited to the generation and manipulation of concentrations of either only one drug solution and one buffer or two drug solutions, simultaneously.

The main objective of the 3D μ-CGG design featured in this work is to perform passive fluidic routing and generation of truly-symmetric three-fluid concentration gradients, which the device accomplishes whilst producing a comparable number of fluid concentration dilutions, which due to the initial concentrations of the stock solutions, captured a 4-fold dilution range. The range of possible solution concentrations can be expanded to 2-logs by increasing the starting concentration of the stock antibiotic solutions. For example, inputting a $\sim 10^2$ concentration into the current device would generate $\sim 10^2$ and multiple $\sim 10^1$ concentrations. To expand the concentration range even further however, the design of the 3D μ-CGG microchannel network can be further improved based on the following design guidelines.

One approach to modifying the 3D μ-CGG microchannel network in order to produce a finer resolution concentration dilution outputs is to increase the number of discrete fluidic outlets by expanding the 3D complexity of the microchannel network itself. For example, by designing one additional layer of vertical microchannels comprising microfluidic mixing and splitting nodes in the the current tetrahedrally symmetric layer-based design, the number of discrete fluidic outputs would increase from 13, as is featured in the experimentally characterized prototype, to 22. To highlight this concept, the solids model and analytical development of a 4-layer 3D μ-CGG microchannel network design is presented in Figure S 14.

Furthermore, an additional approach to improving the capabilities of the 3D μ-CGG microchannel network lies in the flexibility in the 3D solids modeling procedure, which renders the design of 3D μ-CGG fluidic networks with more three-dimensionally complex arrangements and symmetric geometries possible. For example, cubic microchannel networks with four fluidic inlets, pentagonal microchannel networks with five fluidic inlets or hexagonal microchannel networks with six

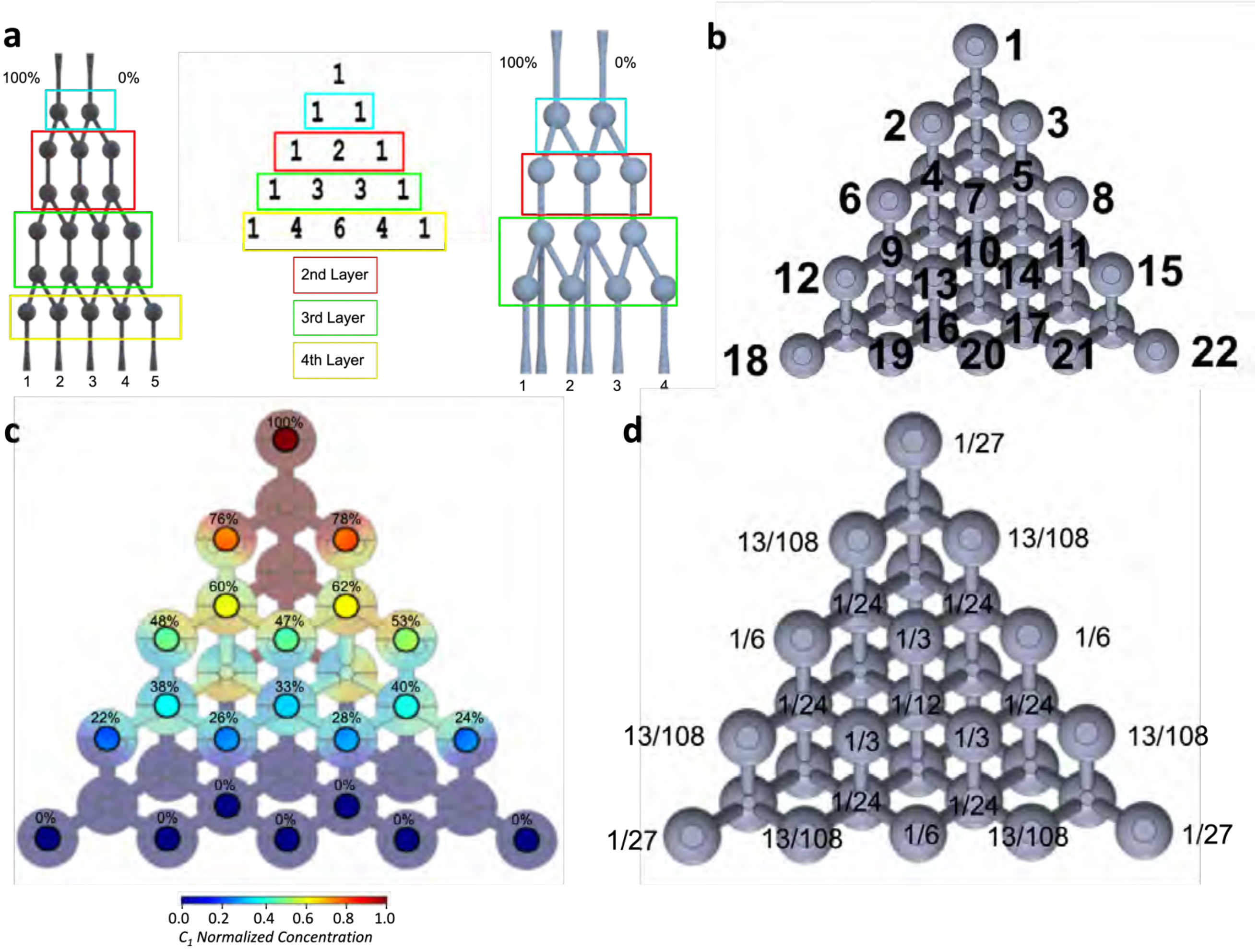

Figure 14. Diagram of the analytical design of a conceptual four-layer 3D μ-CGG network design. **(a)** Layer arrangement. **(b)** Outlet numbering. **(c)** Theoretical concentration of each input concentration at each outlet. **(d)** Analytically-calculated output flow rate at each outlet.

fluidic inlets would generate many more fluidic outlets, enabling the generation of even finer resolution, for example, 3-logs or greater, concentration dilutions. One important consideration that must be taken into account when expanding the 3D geometric complexity of such 3D μ-CGG devices, however, is that more three-dimensionally complex 3D μ-CGG microchannel networks increase the fluidic resistance encountered by fabricated microchannel network prototypes, possibly significantly.

Additionally, the increased complexity and lengths of the microchannel pathways comprising such complex 3D microchannel networks also would increase the difficulty in successfully removing the entirety of the support material from the inside of the hollow microchannel structures. Additional measures to facilitate complete removal of support materials from such larger microchannel networks during post-processing might therefore be necessary to completely post-process such devices, such as the addition of wax-removal inlets on the edges of the device, which would be permanently sealed using epoxy before operation; increasing the hydraulic diameters of the microchannels, which would decrease the overall resistance of the linear microchannels; remove the hollow bulb features as featured in the prototype 3D μ-CGG design demonstrated in this work to reduce the likelihood of residual support material (see Section S1.3 for further discussion); or the implementation of automated pressure-driven mineral oil actuation employing a system such as the Fluigent micro-controller used in this work.

8.2 Approaches to enhancing the functionality of stand-alone and integrated 3D μ-CGG platforms

The experimental setup featured in this work, as shown in Figure S6 was designed and used to accomplish routing of the fluidic outlets from the modular fabricated prototype device to discrete wells on a standard 96-well plate, for ease of off-chip biological incubation and fluorescence image analysis, in order to demonstrate the prototype as a functional proof-of-concept 3D μ-CGG tool. The functionality of the current, as-fabricated prototype as a stand-alone device could be further improved, however, for example in order to better suit point-of-need applications which require more complex fluid handling routines beyond those accomplished using standard 96-well microplate microdilution analysis. Towards this end, a modified experimental setup could be designed and implemented which utilizes outlet tubing lengths which are themselves routed to the inlets to modular microfluidic devices (which were either conventionally or additively manufactured), such as cell culture chambers or bioreactors, in order to deliver the concentration dilutions generated by the 3D μ-CGG device directly to microfluidic hardware for further downstream analysis. In this hypothetical application, a single fabricated 3D μ-CGG device can be used to generate and deliver identical concentration gradients to multiple microfluidic devices for independent analysis which utilize the same concentration gradient profiles, thereby eliminating the need to fabricate separate, identical 3D μ-CGG networks directly into each microfluidic device themselves, thus saving material volume and manufacturing time and cost. Optimization of such an operational setup should be focused on reducing the overall length of tubing required to interface with the device, thereby making the apparatus more compact, and reducing the overall effective fluidic resistance of the network. Such an approach would render the interfacing hardware easier for the operator to operate and allow the hardware to be more portable and travel to different point-of-need settings. Additionally, shortening the overall lengths of the interfacing tubing would allow lower operational flow rates to be used in experiments, allowing dynamic tailoring of the generated concentration gradient to produce flow rate-dependent concentrations at the outlets. Moreover, the throughput of AST applications using additively-manufactured 3D μ-CGG devices can be even more significantly increased by incorporating the 3D gradient generating microfluidic networks into the designs of more complex, monolithically manufacturable 3D printed multi-drug microfluidic micro-total analytical systems (μ-TAS)[61]. These integrated platforms would incorporate 3D μ-CGG microchannel networks into the device itself and enable automated routing of the fluidic outputs from the concentration gradient generation microchannel region to designated regions on the device to perform *on-chip* fluidic routines, such as bacterial incubation and quantification of bacterial proliferation inhibition[13]. By doing so, the various fluidic routines, such as the bacterial enrichment and quantitative detection steps performed *off-chip* in this work can be miniaturized onto future 3D printed 3D μ-CGG-integrated μ-TAS platforms, promising to significantly increase the analytical throughput of numerous biomedical and diagnostic AST applications.

9 Background and introductory material

9.1 Definition of bacterial proliferation

In this work wherever the use of the phrase *bacteria growth* is used, this refers to the increase in the density of bacteria cells in a given volume of liquid bacterial culture media, usually presented in units of colony forming units per milliliter of liquid bacterial stock solution, *cfu/mL*. Here, a single colony forming unit is defined as an individual viable bacterium cell in the given sample, *viable* referring to the ability of each of said cell to reproduce and form a new colony population of cells under the appropriate conditions.[34]. Bacteria, such as *E. coli*, nearly-universally undergo five distinct, well studied, phases of population growth. Bacteria are asexual organisms and reproduce by binary fission. In well-controlled laboratory settings, when a single individual colony forming unit is added to fresh nutrient-rich growth media, after the (*i*) "lag phase" where the organism is adjusting to the chemical dynamics of the new environment, the bacterium begins to reproduce and the population of bacteria increases roughly exponentially, referred to as the (*ii*) "logarithmic phase" of the population growth. In this growth phase, the population of many known strains of *E. coli* doubles approximately every 20 to 30 minutes. Even in initially nutrient-rich

media however, after roughly 12 to 24 hours, the depletion of available nutrients eventually stymies bacterial growth, the rate of cellular reproduction and death roughly equilibrate, and the population growth enters the (*iii*) "plateau phase" or "stationary phase", saturating at approximately 10^8 cfu/mL for roughly 24 hours. The eventual accumulation of toxic byproducts of cellular metabolism causes the population to enter the (*iv*) "death phase", where the population will decrease by two to three orders of magnitude, eventually saturating once more due to the availability of nutrients released from the cellular rupture upon the death of most of the population, and remaining constant during the (*v*) "long-term stationary" phase. Throughout this phase, which can last weeks to months, some of the surviving cells begin to mutate and the viability of most cells in the population is not assured. Therefore when creating a useful bacterial stock for bacteriological experiments, the standard protocol is to create a bacterial inoculation after overnight incubation to use the bacteria population whilst still in the "stationary" phase, where both the consistency of the population density and the viability of the cells are reasonably assured.[62]

9.2 Biophysiological mechanisms behind antibiotic growth inhibition and multi-antibiotic interactions

Antibiotics are chemotherapeutic compounds which are used to treat bacterial infections by inhibiting the growth and replication of pathogenic bacteria, thereby helping the immune system to kill off the remaining bacterial population and fight the infection within the body[63]. The specific mechanisms behind the inhibition of bacterial growth are not perfectly well understood for every antibiotic compound. however with most antibiotics, it is generally-accepted that a single bacterium will metabolically processes an antibiotic molecule, reducing it into a series of intermediate molecules, one or some of which will bind to its DNA and proteins responsible for cell division, inhibiting the bacterium's cell division[39,64]. Bacterial strains do develop antibiotic-resistant bio-physiological pathways through natural selection and genetic mutations, which has been attributed to multiple human-induced factors including the extensive over-prescription of broad-spectrum antibiotics in medicine and in the industrial agriculture industry, patient non-compliance with prescribed antibiotic regimens and a general lack of robust antibiotic stewardship programs in developing nations, in particular[65]. As a result, the efficacy of many clinically-relevant antibiotics is significantly diminished worldwide towards treatment of many different antibiotic-resistant bacterial infections. Conventional antibiotic combination drug screening is performed in a similar manner to MIC testing, yet involves bacterial incubation in the presence of solutions containing specific ratios of different antibiotic compounds[35,66] to ascertain the combined effects of the antibiotics; whereby, certain *synergistic* antibiotic combinations are more effective at inhibiting bacterial proliferation than either of the two antibiotics on their own. Additionally, a two-antibiotic combination is defined as *antagonistic* if the combination is less effective at growth inhibition than either antibiotic on its own. Moreover, a two-antibiotic combination is defined as *additive* if the combination of antibiotics exhibits neither combined effect.[44,67]

Despite this rise in antibiotic-resistance, development of new antibiotic compounds has been practically stalled entirely in developed nations, as only two new classes of antibiotics have been developed and introduced in the past three decades, which are themselves only moderately-effective in treating most bacterial infections[68]. The rate of new antibiotic development and significant advances in the management and prevention of infectious diseases was at an all-time high by the middle of the 20th century; however, the late 20th century witnessed drastic decline in interest on part of the pharmaceutical industry in the development of new classes antibiotics, as companies must invest upwards of $800 million and at least 10 years on average in research and development in order to bring a single novel antibiotic compound to market today, which demonstrates a considerably lower rate of return in terms of profit as compared to developing new drugs targeting chronic diseases[69].

AMR is of particular relevance in the context of bacterial antibiotic resistance, which has been attributed to multiple human-induced factors including the extensive over-prescription of broad-spectrum antibiotics in medicine and industrial agriculture, patient non-compliance with prescribed antibiotic regimens and a general lack of robust antibiotic stewardship programs in developing nations[65]. AST methods generally involve collection of a bodily fluid or tissue sample from a patient, isolating and enriching the infecting pathogen using laboratory-standard protocols, followed by bacterial culture in the presence of antibiotics to assess biological resistance characteristics to different antibiotic chemicals and dosages. These results are then used to better inform clinicians as to the most effective antimicrobial compounds and doses to combat both pre-existing and emerging AMR-related infections[70,71].

In practice, different strains of bacteria evolve varying degrees of sensitivity to certain concentrations of antibiotic compounds at different rates; thus, the nature of clinical AST is such that whenever a definitive evaluation of antibiotic sensitivity is desired, the most medically-rigorous course of action is to perform AST *on-site* in the clinical setting on a sample of that particular bacteria using specific antibiotics available at hand (*i.e.* a patient is admitted to a hospital with symptoms of a suspected type of infection, a bodily fluid sample is collected, and the healthcare professional would like to confirm the appropriate dose of the most effective antibiotic before administering the treatment, taking caution not to inadvertently prescribe an ineffective treatment or prolong patient hospitalization). This course of action is particularly commonplace in the example of the treatment of urinary tract infections (UTI's) caused by strains of bacteria such as *Escherichia coli* (*E. coli*), *Escherichia faecalis* (*E. faecalis*), *Klebsiella pneumoniae* (*K. pneumoniae*),*Staphylococcus aureus* (*S. aureus*) and *Proteus mirabilis* which demonstrate varying degrees of sensitivity to most commonly-administered antibiotics. In suspected cases of UTI outbreaks,

especially in low resource areas, clinical AST evaluation is routinely performed on urine samples in order to identify the particular strain of bacteria by its sensitivity profiles to single antibiotics and to determine the most effective dose of each antibiotic which could be used to treat the infection. In these AST methods, the degree of sensitivity to individual antibiotics, as indicated by the inhibition of bacterial proliferation in a cultured sample in the presence of different concentrations of the given antibiotic, is typically separated into three categories: *Resistant*, *Intermediate Resistance* and *Sensitive*. For example, a recent study by Sabir et al.[72] revealed that out of 500 clinical samples taken from patients with suspected cases of UTI's in a developing nation (performed using the disk diffusion method), *E. coli* was the most prevalent bacteria identified and demonstrated the highest resistance to penicillin (100% of samples tested) and amoxicillin (100%), an intermediate resistance to tetracycline (69.4%), doxycycline (66.6%) and ciprofloxacin (54.2%), and high sensitivity to treptomycin (30%), kanamycin (19.9%) and amikacin (12.7%). Therefore, UTI treatment in this instance with kanamycin or amikacin, which are both of the same family[51], would be the only advisable treatment. Moreover, since ∼81% of the samples tested in this study were resistant to multiple classes of antibiotics, this study recommends testing combinations of multiple antibiotics to find the most optimal treatment. However as this study discusses, the *E. coli* antibiotic sensitivity profile demonstrated in this instance is different from those evaluated for *E. coli* in other parts of the world. Therefore, this study demonstrates the need for consistent region-specific AST evaluations on a case-by-case basis in clinical settings; thus, the frequency of AST and the need for higher throughput analysis is critically needed.[49,72]

References

1. CLSI. Performance Standards for Antimicrobial Susceptibility Testing Performance Standards for Antimicrobial Susceptibility Testing Suggested Citation. *CLSI document M02-A11* 100–125 (2018).
2. Sweet, E. C. *et al.* 3D Printed Chaotic Mixer For Low Reynolds Number Microfluidics. *2019 20th Int. Conf. on Solid-State Sensors, Actuators Microsystems Eurosensors XXXIII (TRANSDUCERS EUROSENSORS XXXIII)* 2258–2261 (2019).
3. Locascio, L. E. Microfluidic mixing. *Anal. Bioanal. Chem.* **379**, 325–327, DOI: 10.1007/s00216-004-2630-1 (2004).
4. Stroock, A. D. Chaotic Mixer for Microchannels. *Science* **295**, 647–651, DOI: 10.1126/science.1066238 (2002).
5. Sochol, R. D. *et al.* 3D printed microfluidic circuitry via multijet-based additive manufacturing. *Lab on a Chip* **16**, 668–678, DOI: 10.1039/c5lc01389e (2016).
6. Oh, K. W., Lee, K., Ahn, B. & Furlani, E. P. Design of pressure-driven microfluidic networks using electric circuit analogy. *Lab on a Chip* **12**, 515–545, DOI: 10.1039/c2lc20799k (2012).
7. Systems, D. High Definition 3D Modeling, Projet HD 3000. Tech. Rep. 800 (2009).
8. Bhargava, K. C., Thompson, B. & Malmstadt, N. Discrete elements for 3D microfluidics. *Proc. Natl. Acad. Sci.* **111**, 15013–15018, DOI: 10.1073/pnas.1414764111 (2014).
9. Glick, C. C. *et al.* Rapid assembly of multilayer microfluidic structures via 3D-printed transfer molding and bonding. *Microsystems & Nanoeng.* **2**, 16063, DOI: 10.1038/micronano.2016.63 (2016).
10. 3D Systems. VisiJet EX200. *Material Data Sheet* (2009).
11. 3D Systems. VisiJet M3 Crystal. *Material Data Sheet* **1**, 1–7 (2016).
12. Sweet, E. C., Mehta, R. R., Lin, R. & Lin, L. Finger powered, 3D printed microfluidic pumps. *TRANSDUCERS 2017 19th Int. Conf. on Solid-State Sensors, Actuators Microsystems* 1766–1769, DOI: 10.1109/TRANSDUCERS.2017.7994410 (2017).
13. Sweet, E. C., Liu, N., Chen, J. & Lin, L. Entirely-3D Printed Microfluidic Platform for on-Site Detection of Drinking Waterborne Pathogens. *Proc. IEEE Int. Conf. on Micro Electro Mech. Syst. (MEMS)* 79–82 (2019).
14. 3D Systems. Visijet S100. *Material Data Sheet* **1910**, 8–10 (2012).
15. Walsh, M. E., Ostrinskaya, A., Sorensen, M. T., Kong, D. S. & Carr, P. A. 3D-Printable Materials for Microbial Liquid Culture. *3D Print. Addit. Manuf.* **3**, 113–118, DOI: 10.1089/3dp.2016.0007 (2016).
16. Williams, D. F. On the mechanisms of biocompatibility. *Biomaterials* **29**, 2941–2953, DOI: 10.1016/j.biomaterials.2008.04.023 (2008).
17. Temiz, Y., Lovchik, R. D., Kaigala, G. V. & Delamarche, E. Lab-on-a-chip devices: How to close and plug the lab? *Microelectron. Eng.* **132**, 156–175, DOI: 10.1016/j.mee.2014.10.013 (2015).
18. Au, A. K., Lee, W. & Folch, A. Mail-order microfluidics: Evaluation of stereolithography for the production of microfluidic devices. *Lab on a Chip* **14**, 1294–1301, DOI: 10.1039/c3lc51360b (2014).

19. Ngo, T. D., Kashani, A., Imbalzano, G., Nguyen, K. T. & Hui, D. Additive manufacturing (3D printing): A review of materials, methods, applications and challenges. *Compos. Part B: Eng.* **143**, 172–196, DOI: 10.1016/j.compositesb.2018.02.012 (2018).

20. Gale, B. *et al.* A Review of Current Methods in Microfluidic Device Fabrication and Future Commercialization Prospects. *Inventions* **3**, 60, DOI: 10.3390/inventions3030060 (2018).

21. Zhu, F. *et al.* Three-dimensional printed millifluidic devices for zebrafish embryo tests. *Biomicrofluidics* **9**, DOI: 10.1063/1.4927379 (2015).

22. Neves, C. B. *et al.* Ethanol postpolymerization treatment for improving the biocompatibility of acrylic reline resins. *BioMed Res. Int.* **2013**, DOI: 10.1155/2013/485246 (2013).

23. Ngan, C. G. *et al.* Optimising the biocompatibility of 3D printed photopolymer constructs in vitro and in vivo. *Biomed. Mater. (Bristol)* **14**, DOI: 10.1088/1748-605X/ab09c4 (2019).

24. Neches, R. Y., Flynn, K. J., Zaman, L., Tung, E. & Pudlo, N. On the intrinsic sterility of 3D printing. *PeerJ* **4**, e2661, DOI: 10.7717/peerj.2661 (2016).

25. MacDonald, N. P. *et al.* Assessment of biocompatibility of 3D printed photopolymers using zebrafish embryo toxicity assays. *Lab on a Chip* **16**, 291–297, DOI: 10.1039/c5lc01374g (2016).

26. Sochol, R. D. *et al.* 3D printed microfluidics and microelectronics. *Microelectron. Eng.* **189**, 52–68, DOI: 10.1016/j.mee.2017.12.010 (2018).

27. Sweet, E. C., Chen, J. C., Karakurt, I., Long, A. T. & Lin, L. 3D printed three-flow microfluidic concentration gradient generator for clinical E. Coli-antibiotic drug screening. *Proc. IEEE Int. Conf. on Micro Electro Mech. Syst. (MEMS)* 205–208, DOI: 10.1109/MEMSYS.2017.7863376 (2017).

28. Wu, S.-Y., Yang, C., Hsu, W. & Lin, L. 3D printed microelectronics for integrated circuitry and passive wireless sensors. *Microsystems Nanoeng.* **1**, 15013, DOI: 10.1038/micronano.2015.13 (2015).

29. Churski, K. *et al.* Rapid screening of antibiotic toxicity in an automated microdroplet system. *Lab on a Chip* **12**, 1629–1637, DOI: 10.1039/c2lc21284f (2012).

30. Boedicker, J. Q., Li, L., Kline, T. R. & Ismagilov, R. F. Detecting bacteria and determining their susceptibility to antibiotics by stochastic confinement in nanoliter droplets using plug-based microfluidics. *Lab Chip* **8**, 1265–1272, DOI: 10.1039/b804911d (2008).

31. Sigma-Aldrich. In Vitro Resazurin Based Toxicology Assay Kit. *Material Data Sheet* .

32. Promega Corporation. CellTiter Blue Cell Viability Assay recommended controls for resazurin. *Mater. Data Sheet* 608–277.

33. Avesar, J. *et al.* Rapid phenotypic antimicrobial susceptibility testing using nanoliter arrays. *Proc. Natl. Acad. Sci.* **114**, E5787 LP – E5795, DOI: 10.1073/pnas.1703736114 (2017).

34. Ewell, M. D. Counting Bacteria. *Science* **ns-15**, 362–362, DOI: 10.1126/science.ns-15.384.362-a (2006).

35. Wiegand, I., Hilpert, K. & Hancock, R. E. W. Agar and broth dilution methods to determine the minimal inhibitory concentration (MIC) of antimicrobial substances. *Nat. Protoc.* **3**, 163–175, DOI: 10.1038/nprot.2007.521 (2008).

36. Omega Optical. Omega Optical 585BP90 Data Sheet. *Data Sheet* 6–7 (2017).

37. Andrews, J. M. Determination of minimum inhibitory concentrations. *The J. antimicrobial chemotherapy* **48**, 5–16 (2001).

38. European Society of Clinical Microbiology and Infectious Diseases. Determination of minimum inhibitory concentrations of antibacterial agents by broth dilution. *Clin. Microbiol. Infect.* **9**, 1–7, DOI: 10.1046/j.1469-0691.2003.00790 (2003).

39. Saint Ruf, C. *et al.* Antibiotic susceptibility testing of the gram negative bacteria based on flow cytometry. *Front. Microbiol.* **7**, 1–13, DOI: 10.3389/fmicb.2016.01121 (2016).

40. Kidsley, A. K. *et al.* Antimicrobial susceptibility of escherichia coli and salmonella spp. Isolates from healthy pigs in Australia. *Front. Microbiol.* **9**, 1–11, DOI: 10.3389/fmicb.2018.01207 (2018).

41. Sarker, S. D., Nahar, L. & Kumarasamy, Y. Microtitre plate based antibacterial assay incorporating resazurin as an indicator of cell growth and its application in the in vitro antibacterial screening of phytochemicals. *Methods* **42**, 321–324, DOI: 10.1016/j.ymeth.2007.01.006 (2007).

42. Ruangpan, L. Chapter 3. Minimal inhibitory concentration (MIC) test and determination of antimicrobial resistant bacteria. *Lab. manual standardized methods for antimicrobial sensitivity tests for bacteria isolated from aquatic animals environment* 31–55 (2004).

43. Duployez, C., Robert, J. & Vachee, A. Trimethoprim susceptibility in E. coli community acquired urinary tract infections in France. *Med. et Maladies Infect.* **48**, 410–413, DOI: https://doi.org/10.1016/j.medmal.2018.03.010 (2018).

44. Yeh, P., Tschumi, A. I. & Kishony, R. Functional classification of drugs by properties of their pairwise interactions. *Nat. Genet.* **38**, 489–494, DOI: 10.1038/ng1755 (2006).

45. Al-Muharrmi, Z., Rafay, A., Balkhair, A. & Jabri, A. A. Antibiotic combination as empirical therapy for extended spectrum Beta-lactamase. *Oman medical journal* **23**, 78–81 (2008).

46. Oluyemi Omoya, F. & Oluyemi Ajayi, K. Synergistic Effect of Combined Antibiotics against Some Selected Multidrug Resistant Human Pathogenic Bacteria Isolated from Poultry Droppings in Akure, Nigeria. *Adv. Microbiol.* **06**, 1075–1090, DOI: 10.4236/aim.2016.614100 (2016).

47. Watkinson, A. J., Micalizzi, G. R., Bates, J. R. & Costanzo, S. D. Novel method for rapid assessment of antibiotic resistance in Escherichia coli isolates from environmental waters by use of a modified chromogenic agar. *Appl. Environ. Microbiol.* **73**, 2224–2229, DOI: 10.1128/AEM.02099-06 (2007).

48. Eliopoulos, G. M. & Eliopoulos, C. T. Ciprofloxacin in combination with other antimicrobials. *The Am. J. Medicine* **87**, S17–S22, DOI: 10.1016/0002-9343(89)90013-2 (1989).

49. Miranda, E. J. P. D. *et al.* Susceptibility To Antibiotics in Urinary Tract Infections in a Secondary Care Setting in Sao Paulo, Brazil, From Urine Cultures. *Revista do Instituto de Medicina Trop. de Sao Paulo* **56**, 313–324, DOI: 10.1590/s0036-46652014000400009 (2014).

50. Amikacin Sulfate. *Amikacin Sulfate Monogr. for Prof.* 1–23 (2017).

51. Dionex. Analysis of the Aminoglycoside Antibiotics Kanamycin and Amikacin Matches USP Requirements. Application Note 267. *Appl. Note 267* 1–6 (2011).

52. Sutherland, C. A., Verastegui, J. E. & Nicolau, D. P. In vitro potency of amikacin and comparators against E. coli, K. pneumoniae and P. aeruginosa respiratory and blood isolates. *Annals Clin. Microbiol. Antimicrob.* **15**, 1–7, DOI: 10.1186/s12941-016-0155-z (2016).

53. Idexx Laboratories Inc. Microbiology guide to interpreting minimum inhibitory concentration. *Diagn. update* (2019).

54. Yeh, P., Tschumi, A. I. & Kishony, R. Functional classification of drugs by properties of their pairwise interactions. *Nat. Genet.* **38**, 489–494, DOI: 10.1038/ng1755 (2006).

55. Puttaswamy, S., Gupta, S. K., Regunath, H., Smith, L. P. & Sengupta, S. A Comprehensive Review of Present and Future Antibiotic Susceptibility Testing Systems. *Arch. Clin. Microbiol.* **09**, 1–9, DOI: 10.4172/1989-8436.100083 (2018).

56. Malmberg, C. *et al.* A novel microfluidic assay for rapid phenotypic antibiotic susceptibility testing of bacteria detected in clinical blood cultures. *PLoS ONE* **11**, 1–15, DOI: 10.1371/journal.pone.0167356 (2016).

57. Flentie, K. *et al.* Microplate-based surface area assay for rapid phenotypic antibiotic susceptibility testing. *Sci. Reports* **9**, 1–9, DOI: 10.1038/s41598-018-35916-0 (2019).

58. Tang, M. *et al.* A linear concentration gradient generator based on multi-layered centrifugal microfluidics and its application in antimicrobial susceptibility testing. *Lab on a Chip* **18**, 1452–1460, DOI: 10.1039/c8lc00042e (2018).

59. Matsumoto, Y. *et al.* A microfluidic channel method for rapid drug-susceptibility testing of Pseudomonas aeruginosa. *PLoS ONE* **11**, 1–17, DOI: 10.1371/journal.pone.0148797 (2016).

60. Yu, H. *et al.* Phenotypic Antimicrobial Susceptibility Testing with Deep Learning Video Microscopy. *Anal. Chem.* **90**, 6314–6322, DOI: 10.1021/acs.analchem.8b01128 (2018).

61. He, Y., Wu, Y., Fu, J. Z., Gao, Q. & Qiu, J. J. Developments of 3D Printing Microfluidics and Applications in Chemistry and Biology: a Review. *Electroanalysis* **28**, 1658–1678, DOI: 10.1002/elan.201600043 (2016).

62. Pletnev, P., Osterman, I., Sergiev, P., Bogdanov, A. & Dontsova, O. Survival guide: Escherichia coli in the stationary phase. *Acta Naturae* **7**, 22–33 (2015).

63. Dafale, N. A., Semwal, U. P., Rajput, R. K. & Singh, G. N. Selection of appropriate analytical tools to determine the potency and bioactivity of antibiotics and antibiotic resistance. *J. Pharm. Analysis* **6**, 207–213, DOI: 10.1016/j.jpha.2016.05.006 (2016).

64. Shaikh, S., Fatima, J., Shakil, S., Rizvi, S. M. D. & Kamal, M. A. Antibiotic resistance and extended spectrum betalactamase types, epidemiology and treatment. *Saudi J. Biol. Sci.* **22**, 90–101, DOI: 10.1016/j.sjbs.2014.08.002 (2015).

65. Cars, O., Hedin, A. & Heddini, A. The global need for effective antibiotics, Moving towards concerted action. *Drug Resist. Updat.* **14**, 68–69, DOI: https://doi.org/10.1016/j.drup.2011.02.006 (2011).

66. Zimmer, A., Katzir, I., Dekel, E., Mayo, A. E. & Alon, U. Prediction of multidimensional drug dose responses based on measurements of drug pairs. *Proc. Natl. Acad. Sci.* **113**, 10442–10447, DOI: 10.1073/pnas.1606301113 (2016).

67. Jain, S. N. & Sampath, A. Antibiotic Synergy Test: Checkerboard Method on Multidrug Resistant Pseudomonas Aeruginosa. *Res. J. Pharm.* **2**, 2011 (2011).

68. Worthington, R. J. & Melander, C. Combination Approaches to Combat Multi-Drug Resistant Bacteria, The problem of multi drug-resistant bacteria. *Trends Biotechnol.* **31**, 177–184, DOI: 10.1016/j.tibtech.2012.12.006 (2012).

69. Conly, J. & Johnston, B. Where are all the new antibiotics? The new antibiotic paradox. *Can. J. Infect. Dis. Med. Microbiol.* **16**, 159–160 (2005).

70. Kim, S., Cestellos Blanco, S., Inoue, K. & Zare, R. Miniaturized Antimicrobial Susceptibility Test by Combining Concentration Gradient Generation and Rapid Cell Culturing. *Antibiotics* **4**, 455–466, DOI: 10.3390/antibiotics4040455 (2015).

71. Wilkerson, C., Samadpour, M., Van Kirk, N. & Roberts, M. C. Antibiotic Resistance and Distribution of Tetracycline Resistance Genes in Escherichia coli O157H7 Isolates from Humans and Bovines. *Antimicrob. Agents Chemother.* **48**, 1066–1067, DOI: 10.1128/AAC.48.3.1066-1067.2004 (2004).

72. Sabir, S. *et al.* Isolation and antibiotic susceptibility of E. coli from urinary tract infections in a tertiary care hospital. *Pak. J. Med. Sci.* **30**, 389–392, DOI: 10.12669/pjms.302.4289 (2014).
